# Bifenthrin Under Scrutiny: Revisiting Toxicological Evidence Amid Regulatory Gaps

**DOI:** 10.1002/jat.4929

**Published:** 2025-09-17

**Authors:** Caroline V. L. Moreira, John Ogbu, Kadja L. C. Monteiro, Thiago M. de Aquino, Edeildo F. da Silva‐Júnior, Alberto S. S. Filho, Hamilton B. Napolitano, Christianah A. Elusiyan, Renê O. do Couto, Elson A. Costa, James O. Fajemiroye

**Affiliations:** ^1^ Laboratory of Pharmacology of Natural and Synthetic Products, Institute of Biological Sciences Federal University of Goiás Goiânia Goiás Brazil; ^2^ Laboratory of Synthesis and Research in Medicinal Chemistry, Institute of Chemistry and Biotechnology Federal University of Alagoas Maceió Alagoas Brazil; ^3^ Research Group in Biological and Molecular Chemistry, Institute of Chemistry and Biotechnology Federal University of Alagoas Maceió Alagoas Brazil; ^4^ Graduate Program in Pharmaceutical Sciences Evangelical University of Goiás Anápolis Goiás Brazil; ^5^ Drug Research and Production Unit, Faculty of Pharmacy Obafemi Awolowo University Ile‐Ife Nigeria; ^6^ Federal University of São João del‐Rei, Midwest Campus Dona Lindu Divinópolis Minas Gerais Brazil

**Keywords:** bifenthrin, biological effects, pyrethroids, regulation, safety, toxicity

## Abstract

Despite growing health concerns, bifenthrin (BF) remains widely used for controlling agricultural and residential pests. However, different perspectives on its toxicological profile and regulatory framework warrant a revisit and update on BF regulation towards a robust risk‐safety assessment. Out of 4102 search outputs, only 62 studies on BF disposition and biological effects met screening and eligibility criteria. Our findings demonstrate oral, dermal, and inhalation toxicity of BF. Studies suggest potential effects, including immunotoxic, endocrine, and reproductive effects, but lack multi‐level substantiation and modeling approaches amidst risk biases. Emerging evidence of anxiogenic, depression‐like, and Parkinsonian‐like effects, which implicate monoaminergic, metabolic, and enzymatic pathways, supports the involvement of several molecular culprits beyond voltage‐gated channel disruption. Toxicity concerns raised by the Food and Agriculture Organization—World Health Organization (FAO/WHO), the United States Environmental Protection Agency (USEPA), and the European Food Safety Authority (EFSA) inform the establishment of reference doses of 0.01, 0.03, and 0.031 mg/kg per day, respectively. While the USEPA considers the current toxicological and safety benchmarks adequate for approved uses, it identified potential acute risks of concern to pollinators and terrestrial invertebrates, leading to precautionary labeling measures. In contrast, the EFSA has questioned the reliability of toxicological data and recommended lowering maximum residue levels. Regulatory disparities are traceable to variations in the study designs and risk assessments. While emerging data on behavioral and neurochemical changes may warrant a regulatory reevaluation of BF's toxicological data, integrating predictive models and other new approach methodologies, using high‐quality designs, may address current regulatory gaps and inconsistencies.

## Abstract 2

Despite health concerns, bifenthrin (BF) remains widely used. However, different perspectives on its toxicological profile and regulatory framework warrant a revisit and update of its regulation to achieve a robust risk‐safety assessment. This review screened 4102 records, identifying 62 studies that met the criteria for BF's disposition and biological effects. The varied study designs and assessments contribute to regulatory inconsistencies, underscoring the need for the application of high‐quality designs to address regulatory gaps.

## 1 Introduction

Bifenthrin (BF) is a Type 1 synthetic pyrethroid that accounts for over 25% of commercially used pesticides worldwide (Deanovic et al. 2018). Its extensive application is underscored by its substantial purchase volume, estimated to exceed 1 million pounds of active ingredient (USEPA 2020). BF's physicochemical characteristics, including high hydrophobicity and photostability (Table S1), contribute to its environmental persistence. These properties and broad‐spectrum applications facilitate the accumulation of its residue in diverse environmental matrices, such as soil, air, and water (Budd et al. 2020; Li et al. 2018; Sanders et al. 2018; Yang et al. 2018). The resulting environmental ubiquity inadvertently creates human exposure pathways and health risks (Corcellas et al. 2012; Liang et al. 2022; Yoshida et al. 2021). Although regulatory frameworks have been developed to ensure its safe use, emerging investigations, spurred in part by BF's growing relevance in green chemistry and nonagricultural sectors, have highlighted its potential toxicological effects (Gargouri, Yousif, Attaai, et al. 2018; Morgan et al. 2018). Findings from these studies underscore the need to revisit the toxicological database from a risk‐safety perspective.

The continued safe use of BF remains a global regulatory concern (Yang et al. 2018). Toxicological assessments conducted by the Food and Agriculture Organization—World Health Organization (FAO/WHO), United States Environmental Protection Agency (USEPA), and the European Food Safety Authority (EFSA) have categorized BF toxicities based on globally accepted standards (Table S2). These authorities establish safety thresholds using toxicological reference values such as acute and chronic reference doses, population‐adjusted doses, and acceptable daily intake. These are derived by applying appropriate uncertainty factors to critical points of departure, as established by these bodies. The acceptable daily intake and reference dose for BF are reported as < 0.02 mg/kg body weight and 0.031 mg/kg per day, respectively (FAO 2009; USEPA 2021; EFSA 2020). The USEPA approves BF use in crops, residential, commercial, pet products, and mosquito control. However, the European Commission banned its application as a plant protection product in 2019, citing concerns about environmental risks. It is currently limited to biocidal use for wood preservation in the European domain (EFSA 2020). Thus, safety issues remain a crucial rationale for evaluating its biological effects and health risks.

Risk assessments inform regulatory safety limits, such as maximum residue levels (MRLs), which represent the estimated daily exposure to a chemical that is unlikely to result in adverse health effects over a defined exposure period (Abadin et al. 2007). Considering that dietary exposure is expected from the use of BF, the USEPA establishes tolerance levels by using exposure risk models that incorporate percent‐crop‐treated estimates and agricultural usage data provided by the U.S. Department of Agriculture. The EFSA reviews available data on residue occurrence, Codex Alimentarius standards, and Member State authorizations to determine food MRLs. However, conclusions drawn from a recent targeted review by EFSA prompted its recommendation to re‐evaluate existing MRLs and the withdrawal of toxicological reference values amid the current restriction (EFSA 2023). The EFSA expressed concerns about insufficient risk assessment and a lack of biological data (EFSA 2020). These disparate regulatory positions underscore the need to review BF's toxicological studies.

Extensive literature review and predictive modeling are recognized as current strategies for integrating toxicological data into risk evaluation (Watford et al. 2019). Despite the growing scientific interest in BF, few reviews offer only fragmented insights into its biological effects (Wolansky and Harrill 2008; Ravula and Yenugu 2021; Yang et al. 2018), lacking systematic integration of regulatory perspectives with a weight‐of‐evidence (WoE) approach. For example, Wolansky and Harrill (2008) critically analyzed the neurobehavioral effects of pyrethroids. However, their review was limited to studies published up to 2008 and focused solely on a single type of biological effect—neurotoxicity. Yang et al. (2018) reviewed the environmental fate and toxicity of BF and its metabolites. Ravula and Yenugu (2021) comprehensively reviewed the chemical and biological aspects of pyrethroid‐based pesticides. However, the lack of regulatory perspectives and WoE evaluation encompassing key risk assessment components—hazard characterization, dose–response, and exposure risk—constitutes a significant knowledge gap. This deficiency is critical given BF's broad‐based application and surrounding regulatory landscape. A comprehensive risk assessment of BF should consider WoE factors, including the quality of study design, consistency of findings across various levels of biological organization and models, and the biological relevance of potential harmful effects to humans. Our review, therefore, examines evidence of BF biological activities while incorporating findings from guideline studies to assess its health risk.

## 2 Methodological Approach

### 2.1 PECO Statement, Research Question, and Selection

This systematic review was prepared from peer‐reviewed articles and guideline studies that addressed the biological activities, toxicity, and underlying mechanisms of BF. A population, exposure, comparator, and outcome (PECO) statement was developed and used to formulate research questions: What is the disposition (O) of BF following exposure (E) in humans and animals (P) compared to the population exposed to low or no BF levels (C)? What are the biological effects and underlying mechanisms (O) of BF (E) in humans and animals (P) compared to unexposed individuals (C)? Do reported BF (E) outcomes (O) present concerns for human (P) health and safety both in the exposed and unexposed populations (C)?

Our review employed search terms with well‐defined inclusion and exclusion criteria grounded in the PECO framework (Table S3). The PECO statement provides information that has been evaluated in human, animal, and in vitro studies. The review of human exposure parameters measured BF or its metabolites, whereas the exposure of animal and in vitro populations measured the administration of BF at varying levels and durations. The control group (or those with low/no exposure) served as the comparator in this review. Considering the scope of studies identified as eligible, we organized these outcomes into BF disposition and biological activities.

**FIGURE 1:**
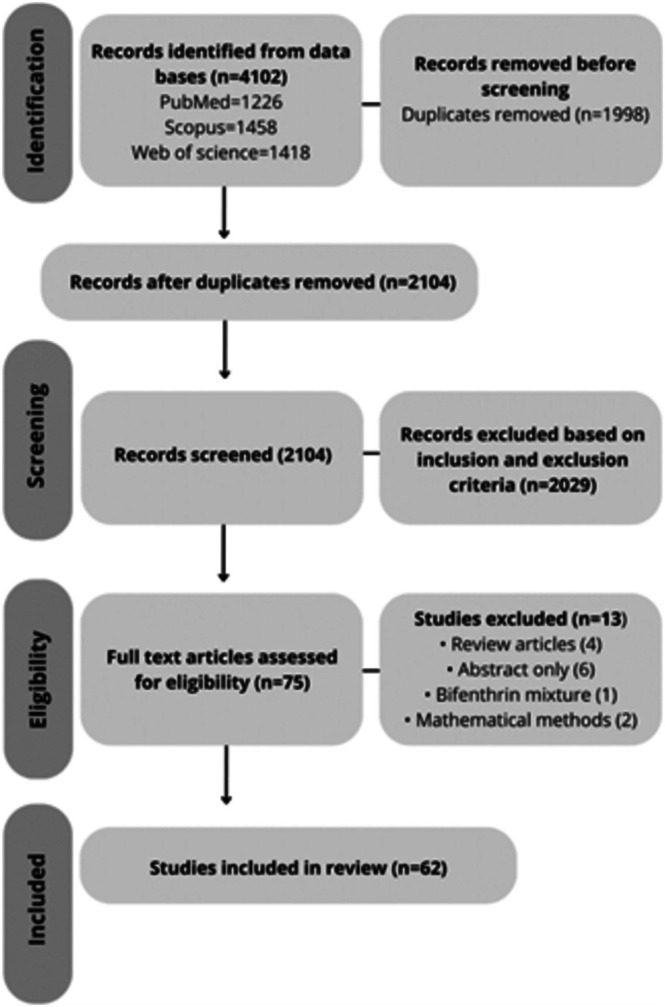
Flowchart diagram of the study.

As shown in the flowchart diagram in Figure 1, relevant scholarly sources (PubMed, Web of Science, and Scopus) were exhaustively queried iteratively using keywords including but not limited to ‘biological activities’, ‘mechanisms’, and ‘toxicity’ combined with ‘bifenthrin’. To ensure a comprehensive search, search terms were meticulously developed. These terms include the following: “synthesis,” “Bifenthrin and biological effects,” “Bifenthrin and toxicity,” “Bifenthrin mechanisms,” “Bifenthrin and pharmacokinetics,” “Bifenthrin and toxicokinetics,” “Bifenthrin and human effects,” “Bifenthrin and rats,” “Bifenthrin and mice.” Our search was limited to articles published from inception till April 1, 2025.

After importing the initial search results into Zotero's standard reference manager, duplicates were removed automatically and manually. The eligibility criteria provided in Table S4 were used to screen articles after removing duplicates. Additional references were hand‐searched from identified articles to capture those missing from the database search. We included studies on chemical and biological effects as well as human, animal, and in vitro‐related studies while excluding irrelevant ones such as those on plant species, with only abstracts available, and non‐English peer‐reviewed literature. Reviewers screened the titles and abstracts of the studies using the PECO framework and eligibility criteria with the Rayyan QCRTI tool. The included studies reported BF disposition, cellular, immunotoxic, neurotoxic, endocrine, reproductive, and miscellaneous activities at both preclinical (Table S5) and clinical (Table S6) levels.

### 2.2 Bias Risk Assessment

Quality is crucial to the WoE evaluation of studies in relation to safety. A risk of bias evaluation of all included studies was conducted according to the Office of Health Assessment and Translation (OHAT) tool guidelines for human and animal studies. According to the National Toxicology Program (NTP) on Extending a Risk‐of‐Bias Approach to in vitro studies, a parallel approach used in human and animal studies was adapted to bias risk assessment for in vitro studies (NTP 2015). According to the NTP‐OHAT systematic review guidelines (NTP‐OHAT 2019), none of the relevant recommendations regarding the use and application of the risk‐of‐bias tool were revised from the 2015 guidance document. The OHAT tool utilizes several risk‐of‐bias questions or domains, including selection, performance, attrition/exclusion, detection, and selective reporting bias, among others, in both preclinical (Table S7) and clinical (Table S8) studies. Two reviewers independently evaluated the bias risk in our systematic review, and the review team discussed any incongruities. The response option for every question was rated by using “‐‐” for “definitely high risk of bias,” “‐” for “probably high risk of bias,” “+” for probably low risk of bias, and “++” for “definitely low risk of bias.” The lower the risk of bias, the higher the study's quality.

After a critical appraisal of the internal validity of each study, reviewers used the OHAT's tiering system to characterize the overall risk of bias score based on three key domains: confounding (for human studies), exposure, and outcome. Studies rated as “definitely low” or “probably low” risk of bias for these key elements were categorized as Tier 1. Studies rated as “definitely high” or “probably high” risk of bias for these key domains were categorized as Tier 3. Studies that met neither Tier 1 nor Tier 3 criteria were classified as Tier 2.

### 2.3 Guideline Studies on Toxicology and Risk Assessment

Finally, we summarized toxicological and risk assessment data from the USEPA, EFSA, and FAO/WHO to analyze the health risk of BF (Tables S9–S14). Using a WoE approach, we examined the quality and consistency of evidence in the included studies, with established guidance values for critical endpoints reported in these data.

## 3 Results

### 3.1 Outcome of Research Question and Selection

We initially found 4102 results in our database search, but after removing 1998 duplicates, we were left with 2104 records. Upon reviewing the titles and abstracts based on our inclusion and exclusion criteria, we identified 2029 articles that did not meet our criteria. The remaining articles were further screened, and 13 were removed because they were review articles, had only abstracts, involved BF mixtures, and utilized mathematical methods. As a result, 62 studies (1.5% of the total references and citations) were included in our review, as shown in Figure 1.

### 3.2 Risk‐Of‐Bias Evaluation and Guideline Studies on the Risk Assessment

After two reviewers independently assessed the risk of bias, 59 preclinical studies were rated for their risk of bias. No study was rated as Tier 3 for risk of bias in any of the studies. According to the OHAT approach, 26 studies were assigned to the Tier 1 category, while 33 were assigned to the Tier 2 category, based on the key domains. About 23 (40%) preclinical studies failed to adequately characterize the exposure domain in terms of purity and concentration. These studies were rated “probably or definitely high risk of bias” for exposure characterization due to missing data on the percentage purity of BF, unconfirmed concentration, and stability/homogeneity of the dosing solution. Twenty studies were rated as low‐risk for outcome bias according to the assessment.

Three human studies were assessed for their risk of bias, particularly in key domains (confounding and outcome biases). One was assigned to the Tier 1 category, while two were categorized as Tier 2. The Tier 2 studies that reported BF's disposition failed to account for confounding factors. Yoshida et al. (2021) did not analyze for any potential confounders, such as children's sex and age, in relation to the presence of BF metabolite in urine after inhalation exposure. Although Corcellas et al. (2012) reported that maternal age did not correlate with BF levels in breast milk, these authors did not account for other potential covariates, such as parity and postpartum body mass index. The data presented in Tables S9–S14 from guideline studies on toxicology and risk assessment of BF to human health were integrated into our WoE evaluation of the included studies. They facilitated our findings under the disposition and effects of BF (Sections 3.2.1 and 3.2.2, respectively).

### 3.3 Bifenthrin Disposition and Toxicity

#### 3.3.1 BF Disposition

Preexposure factors (such as dose and routes of administration) and the fate of BF in the body after exposure have significant health implications. Our search output identified nine studies that evaluated the absorption, distribution, metabolism, and excretion of this pyrethroid to determine its disposition. The solubility, time course, or concentration‐time relationships could provide valuable information on the effects of BF, risk potential, and biomonitoring approaches.

Exposure to the pyrethroid occurs via oral, dermal, and inhalation routes. Different studies have reported an oral absorption rate of between 40% and 60%, as evidenced by its presence in blood, brain, liver, and adipose tissue (Gammon et al. 2014; Hughes et al. 2016; Wolansky et al. 2007). Dermal absorption is considerably lower. Hughes and Edwards (2010) estimated dermal absorption at < 10% and reported that post‐exposure skin cleansing, performed 24 h after BF exposure, can eliminate up to 83% of the compound. This suggests a poor transdermal uptake under standard exposure scenarios. Although less frequently examined, inhalation is an exposure route. Two studies (1 animal and 1 human) demonstrated evidence of BF exposure from the inhalation route (Gammon et al. 2014; Yoshida et al. 2021). In a study of Japanese children (*n* = 132) aged 6–15 years, inhalation contributed 0.084% of total BF exposure while accounting for 15% of the plasma concentration (Yoshida et al. 2021). The absence of the first‐pass effect after inhalation exposure provides a rationale for investigating the pharmacokinetics of BF following this exposure route. BF concentration after inhalation was approximately 75%–80% of that observed after oral administration.

Following absorption into the systemic circulation, BF achieves an oral bioavailability < 40% with significant concentration in fat stores (Hughes et al. 2016). The distribution of BF into the liver, brain, and adipose tissue was supported by its intravenous administration. At an oral dose of 3 mg/kg, the concentration of BF was 36%–100% higher in the liver than in the blood. Importantly, BF crosses the blood–brain barrier, with brain concentrations reaching 11.9 and 143.2 ng/mL following oral doses of 0.3 and 3.0 mg/kg, respectively, reinforcing concerns about potential neurotoxicity (Hughes et al. 2016). Its concentration‐time course is crucial for evaluating its bioaccumulation potential, a significant risk assessment parameter. The blood concentration of BF peaks within 1–2 h after absorption (Table 1). Intravenous administration demonstrated a two‐phase kinetic disposition characterized by a rapid distribution phase (*t*_½_ = 0.17 ± 0.08 h, mean ± SD) and a slower elimination phase (Gammon et al. 2014; Hughes et al. 2016).

**TABLE 1:** Pharmacokinetic parameters of bifenthrin.

Study	Route of administration/animal	Dose	Sites of internal exposure	Pharmacokinetic parameters			
				*C*_max_ (ng/mL)	*T*_max_ (h)	*T*_1/2_ (h)	AUC (ng.h/mL)
Hughes et al. 2016	Oral/Long‐Evans rat	0.3 or 3.0 mg/kg	Blood	86.1 or 946.5	1–2	2.8 or 2.9	702.4 or 642.6
			Brain	11.9 or 142.8	4 or 6	13.9 or 11.6	677.5 or 925.1
			Liver	83.7 or 1146.3	1 or 2	2.9 or 13.9	957.1 or 1264.1
			Adipose	157.1 or 1445.3	8 or 24	1.0 × 10^−3^ or 1.0 × 10^−3^	1.7 × 10^5^ or 1.8 × 10^5^
	Intravenous/Long‐Evans rat	0.3 mg/kg	Blood	—	—	8.2	1077.6
Gammon et al. 2014	Oral/Sprague–Dawley rat	3.1 mg/kg	Plasma	361.0	2	—	1969.0
			Brain	83.0	8	—	763.0
	Inhalation/Sprague–Dawley rats	0.018 mg/kg	Plasma	232.0	2	—	1584.0
			Brain	73.0	12	—	619.0
	Intravenous/Sprague–Dawley rats	1.0 mg/kg	Plasma	889.0	—	13.4	453.0
			Brain	95.0	6	11.1	1303.0
Gammon et al. 2012	Oral ^14^C‐bifenthrin/Sprague–Dawley rats	6.1 mg/kg	Plasma	664.0	4–6	11–12	—

Abbreviations: AUC: The area under the concentration‐time curve; *C*_max_: Maximum (peak) concentration; *T*_max_: Time taken to reach maximum concentration; *T*_1/2_: Half‐life.

**FIGURE 2:**
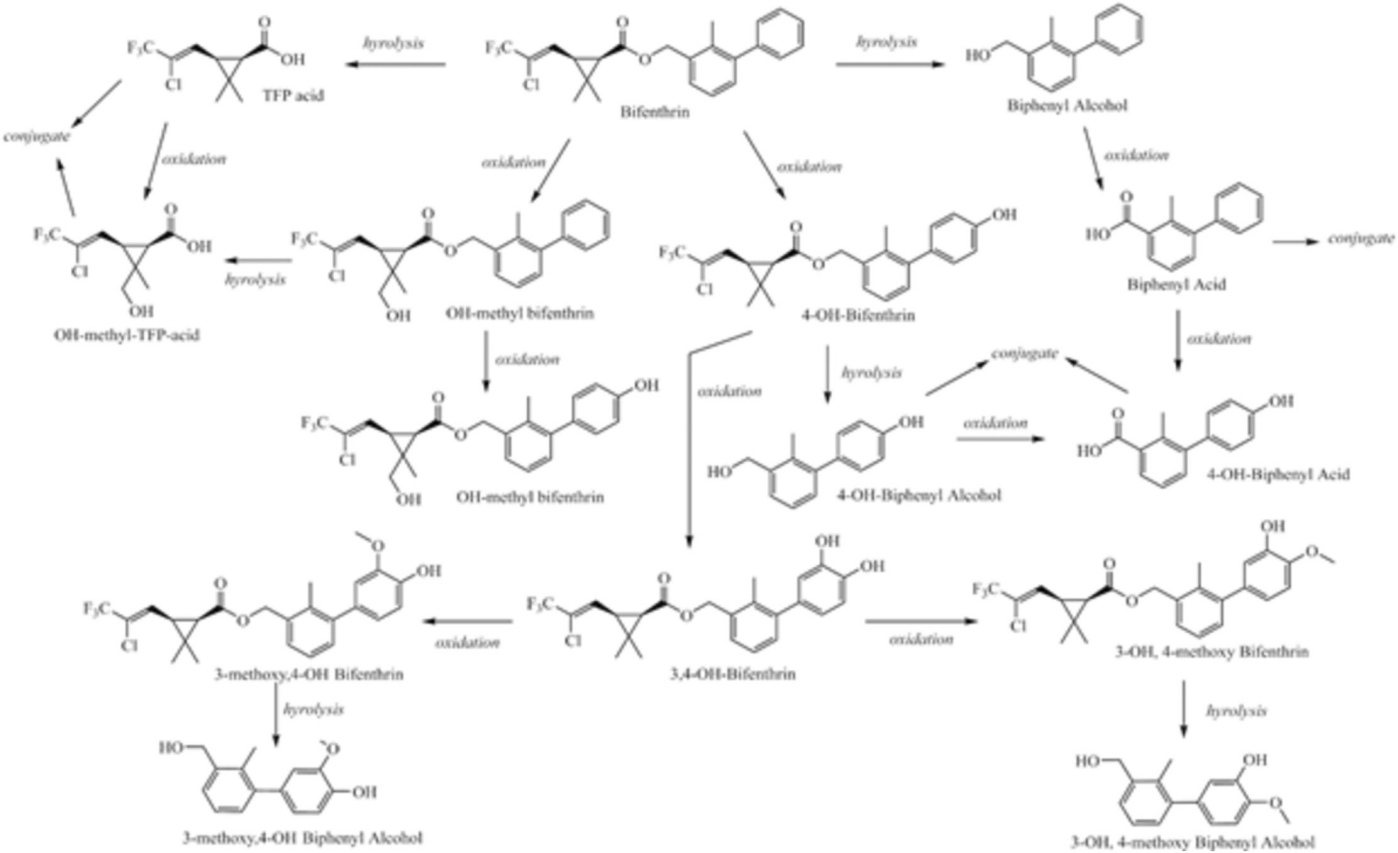
Bifenthrin metabolic pathways.

Metabolic transformation of BF is predominantly hepatic and biphasic (Treinen‐Moslen 2001). Phase I metabolism involves rapid oxidation (~80%) primarily through cytochrome P450 enzymes, as well as the hydrolysis of the ester bond (Figure 2). These reactions lead to the generation of key metabolites, including 4′‐hydroxy‐bifenthrin, formed through oxidative and hydrolytic pathways (Gammon et al. 2012; Liu et al. 2005; Nallani et al. 2018; USEPA 2020). Phase II metabolism, though less thoroughly characterized, involves conjugation reactions, facilitating the excretion of BF and its metabolites. Although interindividual variability in metabolic rates, particularly age‐related differences in enzymatic activity, has been reported (Nallani et al. 2018), these findings are largely derived from in vitro models and still lack sufficient in vivo validation.

Pyrethroids taken into the body via several routes are excreted in feces and urine. BF is mainly excreted in feces (USEPA 2020). One study reported the presence of BF in the urine of Japanese children (Yoshida et al. 2021). Its elimination half‐life depends on the rate of decline in blood concentration. Oral administration of 0.3 and 3 mg/kg doses resulted in a substantially longer elimination half‐life for the lower dose, 3 times that of the higher dose. While BF is rapidly cleared from the blood and liver, it demonstrates prolonged persistence in the brain and adipose tissue, with half‐lives of approximately 14 and 21 days, respectively (Hughes et al. 2016). Following the intravenous administration of 3 mg/kg BF, the blood BF concentration decreased biexponentially, with an elimination half‐life of 8 h. In rats administered an intravenous dose of 1 mg/kg of BF, the blood elimination half‐life was 13.4 h. The decline in blood levels of pesticides corresponds with the reversal of clinical signs of neurotoxicity (Gammon et al. 2012, 2014).

#### 3.3.2 General (Acute and Chronic) Toxicity of Bifenthrin

Evaluating the toxicity of BF is crucial for understanding its potential health risks. Clinical signs of toxicity, including tremors, convulsions, jerks, and twitches, have been associated with BF exposure. Acute, sub‐chronic, and chronic toxicity data assessed by regulatory bodies are summarized in Table S9. The USEPA analyzed various guidelines and non‐guideline (special) studies to assess the risk of BF to human health. Following an acute oral toxicity study in rats, BF demonstrated a LD_50_ of 58.4 and 43.0 mg/kg in males and females, respectively. The FAO/WHO recorded a LD_50_ of 53.4 mg/kg for acute oral (rat) toxicity. Based on guideline assessments of acute dermal, inhalation, eye, and skin irritation, BF is categorized as Toxicity Category III for acute dermal and eye exposure routes and Toxicity Category IV for skin irritation potential. According to Classification, Labelling, and Packaging criteria, BF is ruled as R25 (toxic if swallowed) based on the LD_50_ of 54.5 mg/kg (in diluted corn oil) and 186.1 mg/kg (in undiluted corn oil) obtained from an acute oral toxicity study. In the acute (dermal) study, BF showed the LD_50_ > 2000 mg/kg, similar to the EFSA assessment. This finding aligns with those of the EFSA and FAO/WHO. In an acute inhalation assessment, the LD_50_ values of 1.10 and 0.80 mg/L were obtained for males and females, respectively. The joint FAO/WHO recorded an LC_50_ of 0.8 mg/L, dust (4 h exposure, nose only). According to the Classification, Labeling, and Packaging regulation criteria for acute inhalation toxicity, BF is ruled as R23 (Toxic by inhalation), based on a rat LC_50_ inhalation of 1.01 mg/L/4 h (CI: 066–1.1) obtained from a toxicity study. However, acute toxicity studies for skin and eye irritation were ruled nonirritant.

Chronic toxicity studies were conducted for BF using tremor incidence as a critical effect; the no‐observed‐adverse‐effect level (NOAEL) and lowest‐observed‐adverse‐effect level (LOAEL) were determined. In a 90‐day oral toxicity study, BF recorded a NOAEL of 3.8 and 4.3 mg/kg per day in males and females, respectively. A study using dogs recorded an LD_50_ of 2.21 mg/kg per day for BF in males and females. In a dermal toxicity assessment in male and female rabbits, the NOAEL and LOAEL of 88 and 442 mg ai/kg per day (based on loss of muscle coordination and increased incidence of tremors), respectively, were recorded. In an inhalation toxicity assessment in male and female rats, NOAEL and LOAEL of 0.0059 and 0.0196 mg/L/day (based on increased tremors and increased respiration rate) were observed, respectively, based on loss of muscle coordination and increased incidence of tremors. Generally, repeated dosing of animals with BF did not increase toxicity higher than that observed in acute exposure.

The USEPA considered some non‐guideline or special studies in its risk evaluation. These special studies provide advantages in utilizing the benchmark dose (BMD) approach, beyond the traditional NOAEL method. Several BMD values were determined, including the BMD and its lower limit corresponding to a 20% increase in neurobehavioral endpoints following a single acute dose (BMD20 and BMDL20, respectively), as well as the BMD and its lower limit for a one standard deviation response (BMD_1sd_ and BMDL_1sd_, respectively). Weiner et al. (2009) reported BMDL20 and BMD20 values of 0.4 and 14.3 mg/kg, respectively, based on multiple functional observatory battery changes after rats were exposed to 0, 40, and 55 mg/kg via gavage in 5 mL/kg of corn oil. In Long Evans rats exposed to BF (0–28 mg/kg via gavage in 1 mL/kg of corn oil), the pyrethroid showed BMD_1sd_ and BMDL_1sd_ of 4.1 and 3.1 mg/kg, respectively, based on decreased locomotor activity (Wolansky et al. 2006). Conversely, this study by Wolansky et al. (2006) was not available to the EU peer review (EFSA 2023).

In addition, decreased locomotor activity (significant at doses ≥ 4 mg/kg) was the most sensitive endpoint in the study by Wolansky et al. (2006), leading USEPA to select this measure for acute dietary and short‐term incidental oral risk assessments. While muscle tremors were noted in all experimental studies, motor activity was not evaluated in every study, indicating a weakness of unselected studies. Wolansky et al. (2006) employed a sensitive rat strain and administered gavage dosing using a vehicle and volume that elicited the most adverse responses (i.e., 1 mL/kg corn oil), which were significant advantages compared to other studies. No gender sensitivity was noted, and dermal and inhalation risks were assessed.

### 3.4 Biological Effects and Mechanisms

#### 3.4.1 Cellular Effects of Bifenthrin

Cellular effects, such as cell viability, proliferation, and lactate dehydrogenase release, are crucial to understanding the biological effects of a given exposure (Beghoul et al. 2017; Gargouri, Yousif, Bouchard, et al. 2018). Fourteen studies from our search output primarily reported BF's cellular effects. Peer‐reviewed studies mainly employed metabolism‐based in vitro assays to assess the cellular effects of the pyrethroid. Using a quantitative colorimetric Alamar Blue assay, Tran et al. (2006) demonstrated that at concentrations lower than 10^−7^ M, BF did not affect the viability of PC12 cells after a 1‐h incubation. In another study using the same cell lines and assay, similar findings were observed at concentrations between 10^−9^ and 10^−6^ mol/L, along with a 1.21‐fold increase in lactate dehydrogenase, indicating little to no signs of cytotoxicity at these doses. However, cytotoxicity was observed at concentrations above 10^−6^ mol/L, with a corresponding elevation in lactate dehydrogenase levels, apoptosis, and disorder of F‐actin filaments (Lu et al. 2011).

The MTT assay provides sensitive measurements of normal cellular metabolic status in mitochondria, where measurements reflect early cellular redox changes. In a study employing this assay, Hep G2 cells were incubated with several concentrations (5, 10, 15, and 20 mg/L) of cis‐BF racemate and its enantiomers. Enantioselective reduction in cell viability was observed following exposure to 15 and 20 mg/L of cis‐BF (Liu and Li 2015). Besides cell viability and lactate dehydrogenase release, apoptosis was observed in FL cells following exposure to different concentrations (7.5, 15, 30, and 60 mg/L) of BF enantiomers or racemate for 24 h (Liu et al. 2008). HCT116 cells incubated at 37°C for 24 h with increasing concentrations of BF (5 to 200 μM) demonstrated a marked decrease in cell viability from 97% in the control to 18% at 200 μM (IC_50_ = 32 μM) (Bouaziz et al. 2020). These cellular effects induced by the pyrethroid may be attributed to apoptotic mediators in the cell. A major family of apoptotic proteins is the Bcl‐2 family, which includes Bcl‐2 and Bax proteins. The Bcl‐2: Bax protein ratio has been correlated with cell death (Ouyang et al. 2012). Liu and Li (2015) reported an increase in Bax proteins and a corresponding decrease in Bcl‐2 in Hep G2 cells incubated with BF (10 or 20 mg/L) for 6 h. The increase in BF‐induced activation of mitogen‐activated protein kinases, p38, N‐terminal, and extracellular‐signal‐regulated kinase suggests their role in apoptotic effects (Bouaziz et al. 2020).

Carcinogenicity and mutagenicity pose health risks associated with pesticide exposure. Based on the Organization for Economic Cooperation and Development (OECD) guideline for chemical testing, regulatory bodies assessed these endpoints using in vitro and in vivo assays (Tables S9 and S10). The USEPA ruled BF to be a “possible human carcinogen” based on a mouse study (NOAEL = 6.7 mg/kg per day in males and 8.8 mg/kg per day in females) in which tremors were manifested (USEPA 2020). Similar findings were observed in the assessment of EFSA and the FAO/WHO: EFSA classified BF as Carc. 2, H351 “suspected of causing cancer” based on an 18‐month mouse study that recorded a NOAEL of 7.6 mg/kg bw/day (ECHA 2009). The FAO/WHO recorded a NOAEL of 50 ppm in mice, equivalent to 7.6 mg/kg body weight per day, based on tremors at the LOAEL of 200 ppm, equivalent to 29 mg/kg body weight per day. Similarly, in rats submitted to a long‐term combined study of toxicity and carcinogenicity, tremors were the most prevalent findings in both sexes. In conducting carcinogenicity and genotoxicity studies, the USEPA found no evidence of carcinogenicity based on a long‐term rat study (NOAEL = 3 mg/kg bw per day). At the highest dose of 200 ppm, equivalent to 9.7 mg/kg body weight per day, the FAO/WHO observed a slight decrease in body weight, with equivocal evidence of decreased food consumption. At the highest dose, retinal atrophy was noted in 28 females but not males. The NOAEL was 50 ppm, equivalent to 2.3 mg/kg bw per day, based on the observation of tremors at the LOAEL of 100 ppm, equivalent to 4.7 mg/kg bw per day. There were no treatment‐related neoplastic findings in rats. Therefore, the USEPA and FAO/WHO concluded that BF is unlikely to pose any carcinogenic risk because tumors were not treatment‐related.

In guideline studies, males at the highest dose showed an increased incidence of urinary bladder tumors (leiomyosarcomas), which was statistically significant. Following revision by expert pathologists, the USEPA and FAO/WHO considered the tumor benign, of vascular origin, and unlikely to be relevant to humans, despite some concerns (FAO 2009; USEPA 2020). According to the EFSA, the tumors observed in the exposed mice were multi‐site (urinary bladder, lung, liver, and leukemia). As such, the carcinogenic potential of BF could not be excluded without mechanistic data. It is unclear whether the mechanistic aspects of these guideline studies may have addressed the gaps identified in the FAO/WHO assessments. The liver tumors (observed only in males) were dose‐related and not statistically significantly increased. Based on historical controls, they were considered unlikely to be treatment‐related. Lung tumors were neither dose‐related nor did they show dose trends. A complementary assessment of these studies identified similar submucosa lesions in the controls, thus rendering historical control data unreassuring.

#### 3.4.2 Immunotoxic and Inflammatory Effects

Pesticides are potential immunotoxins that can cause human immune dysfunction (Diel et al. 2003; Kannan et al. 2000; Madsen et al. 1996). Considering the protective role of the immune system, pesticide‐induced compromise of this system can lower innate defenses, making it more susceptible to a wide range of infectious and inflammatory disorders. Three literature studies reported BF‐induced alterations in the immune system. Hoffman et al. (2006) reported a T‐cell LFA‐1/ICAM‐mediated homotypic aggregation in H9 and Jurkat T‐cell lines using pure and commercial BF at concentrations that did not reduce cell viability. In the same study, BF activated the aggregation of T cells, possibly by lymphocyte function‐associated antigen‐1 and intercellular adhesion molecule‐1. Pro‐inflammatory factors, including IL‐1β, IL‐6, and TNF‐α, have been linked to immune system changes following BF exposure. Wang et al. (2017) reported in an in vitro study that the production of IL‐1β, IL‐6, CXCL‐1, and TNF‐α was inhibited in peritoneal macrophages, spleen, and thymus. Contrary to these findings, other reports have shown that BF increases TNF‐α and other pro‐inflammatory cytokines in an in vivo model (Jin et al. 2012, 2014). This discrepancy may be attributed to the different models used in these studies. In addition to changes in pro‐inflammatory cytokines, decreased thymus weight and inhibition of splenocyte proliferation were observed following BF exposure (Jin et al. 2012).

The toxicology database of guideline studies for BF does not indicate any evidence of treatment‐related effects on the immune system, and the overall WoE evaluation suggests that it does not directly target the immune system. The USEPA's study (OPPTS 870.7800) assesses a chemical's immunosuppressive potential by measuring antibody production against sheep red blood cells in mice or rats, relying on the functionality of T‐cells and B‐cells to accurately assess immune function (USEPA 2013). The OECD uses the Extended One‐Generation Reproduction Study (OECD Test Guideline 443) to assess the potential impact of chemical exposure on the developing immune system, serving as a substitute for a guideline study. Together, BF was not considered to be immunotoxic.

#### 3.4.3 Neurotoxic Effects

Neurotoxicity is a significant biological indicator in classification and risk assessment (ECHA 2009). Comprehensive neurobehavioral assessments of neuromuscular, locomotor, and behavioral endpoints have been used to characterize the acute neurotoxic effects of BF. Hyperactivity, tremors, decreased motor activity, reduced grip strength, increased pawing, and head shaking were considered pyrethroid‐like effects (Holton et al. 1997; Wolansky et al. 2007). We identified six preclinical studies that have linked BF exposure to neurobehavioral impairments, including mood and cognitive types (anxiety, depression, motor incoordination, learning, and memory loss). Oral administration of BF (7 mg/kg per day for 30 days; 0.6 or 2.1 mg/kg per day for 60 days) in rats decreased time spent in the open arm and increased time spent in the closed arm of the elevated plus maze, thus suggesting an anxiogenic‐like property (Gargouri, Bhatia, et al. 2018; Abdel‐Wahhab, Sayed, et al. 2024). According to Gargouri, Bhatia, et al. (2018), rats exposed to BF (0.6 or 2.1 mg/kg per day) for 60 days exhibited depressive‐like behavior. Besides anxiogenic‐ and depressive‐like properties, BF induced motor incoordination and memory impairments in exposed rodents (Abdel‐Wahhab, Sayed, et al. 2024; Gargouri, Yousif, Attaai, et al. 2018; Gargouri, Bhatia, et al. 2018; Gomaa et al. 2021; Syed et al. 2016, 2018; Zhang and Zhang 2024).

In addition, BF decreased latency to fall and increased climbing time in pole and rotarod tests in neonates. In a developmental neurotoxicity study using female Sprague–Dawley rats (*n* = 25/group) exposed to dietary BF (0, 50, 100, or 125 ppm), the dams and pups exhibited tremors and clonic convulsions, as well as a slight reduction in acoustic startle response amplitude (Gammon et al. 2019). Syed et al. (2016) reported no significant changes in surface righting and negative geotaxis reflex, though there was impaired neonatal pivoting and locomotion in BF‐exposed rats. During neuronal development, synaptic networks are formed. These networks have been associated with synchronized Ca^2+^ oscillations, which are necessary for neurite development and activity‐dependent growth (Wayman et al. 2006; Cao et al. 2012). Acute exposure to BF rapidly increased the frequency of synchronized Ca^2+^ oscillation by 2.7‐fold (EC_50_ = 58 nM) and decreased its amplitude by 36%, independent of modifications in voltage‐gated channels. Subacute treatment enhanced neurite growth, which may be partly mediated by the activation of metabotropic glutamate receptors (Cao et al. 2014).

Toxicological endpoints in the BF database are consistently based on clinical signs of neurotoxicity, specifically tremors (USEPA 2020). Multiple guideline studies on BF neurotoxicity, encompassing acute, sub‐chronic, and developmental neurotoxicity assessments, were utilized to determine the level of concern and health risk (Table S11). In an acute rat neurotoxicity study in which liquefied BF (heated to 80°C) was administered without a vehicle, the pyrethroid demonstrated a NOAEL of 35 mg/kg and a LOAEL of 75 mg/kg based on mortality for females, findings from a functional observational battery, and differences in motor activity. Sub‐chronic neurotoxicity assessment demonstrated that BF was toxic, having a NOAEL of 50 ppm (equivalent to 2.9 and 3.7 mg/kg per day in males and females, respectively) and a LOAEL of 100 ppm, equivalent to 6.0 and 7.2 mg/kg per day in males and females, respectively. Following the assessment of developmental neurotoxicity, the maternal NOAEL at gestation and lactation was found to be 3.6 and 8.3 mg/kg, respectively. Based on clinical signs of neurotoxicity observed in mothers, a LOAEL of 7.2 and 16.2 mg/kg per day during gestation and lactation was established, respectively. In the developmental neurotoxicity assessment, BF showed a NOAEL of 3.6 and 8.3 mg/kg during gestation and lactation, respectively. Based on increased grooming counts, BF showed a LOAEL of 7.2 and 16.2 mg/kg per day during gestation and lactation, respectively.

In a peer‐reviewed study by Wolansky et al. (2006), which the USEPA and FAO/WHO considered in regulatory assessment (Table S11), male rats were treated orally with BF in 9 doses (8–18 rats per dose) ranging from 0.03 to 28 mg/kg bw in corn oil (1 mL/kg bw) and motor activity was assessed for 1 h during the period of peak effects (4 h after dosing). The data were modeled, and a threshold dose of 1.28 mg/kg bw was determined. The threshold dose estimates the highest no‐effect dose level at which treated rats did not display any significant decreases in motor activity. ECHA conducted several neurotoxicity assessments. Clinical signs of neurotoxicity (tremors, twitching) were observed from 100 ppm in Sprague–Dawley rats administered 0, 50, 100, and 200 ppm of technical BF. In a range‐finding developmental neurotoxicity study, tremors and clonic convulsions at 125 ppm were generally observed, with maternal neurotoxicity recorded at 100 ppm.

The alteration of ion channels, enzymes, ATPases, and neurotransmitter levels, among others, contributes to BF's neurotoxic mechanisms (Yang and Li 2015). The principal target of the neurotoxic action of BF is the neuronal membrane's voltage‐gated Na^+^ channel (Cao et al. 2014). Figure S1 summarizes the main target of BF's toxic action in the cell membrane. A decrease in Na^+^/K^+^‐ATPase and Mg^2+^‐ATPase activities after BF exposure has been linked to behavioral disorders in preclinical studies (Gargouri, Yousif, Bouchard, et al. 2018, 2019). Anxiety, depression, and motor and cognitive impairments may be linked with BF‐induced perturbations of neurotransmitter levels (Gargouri, Yousif, Bouchard, et al. 2018). Decreased monoamine levels were associated with anxiogenic‐like effects and motor incoordination in rodents treated with BF (3.5 and 7 mg/kg per day) for 30 days (Syed et al. 2018; Abdel‐Wahhab, Sayed, et al. 2024). Moreover, evidence suggests that autophagic proteins may be involved in BF's neurotoxic effects. In male Parkin knockout and C57BL/6 mice exposed to BF (10 mg/kg/day) for 28 days, Zhang and Zhang (2024) reported increased expression of mitochondrial autophagic proteins and their receptor. These preclinical findings reinforce neurotoxicity evidence linked to BF.

#### 3.4.4 Endocrine and Reproductive Effects

Seven studies (5 in vitro, 1 animal, and 1 human) evaluating the endocrine effects of BF were identified (Tables S5 and S6). The synthesis and transport of cholesterol rely on intracellular messengers and steroidogenic genes, including 3‐hydroxy‐3‐methylglutaryl‐CoA synthase (HMG‐CoA synthase), the low‐density lipoprotein receptor (LDL‐R), and the peripheral benzodiazepine receptor (PBR). In four‐week‐old adolescent male ICR mice exposed to 0, 7.5, and 15 mg/kg per day BF for 3 weeks, the highest dose (15 mg/kg/day) significantly altered mRNA expression of HMG‐CoA synthase, HMG‐CoA reductase, LDL‐R, and PBR, and reduced serum testosterone levels compared with controls (Jin et al. 2014). The pyrethroid decreased the basal production of cortisol and aldosterone in human adrenocortical H295R cells exposed to 1, 10, and 100 nM BF for 24 h (Yang et al. 2022). BF may downregulate steroidogenic genes and intracellular signaling processes regulating cholesterol synthesis and mitochondrial transport in steroid‐dependent cells and organs, especially at higher doses (Zhang et al. 2016; Zhang et al. 2018).

In addition to hormone production, BF may mimic estrogen activity. Exposure of the human breast carcinoma cell line MCF‐7 to BF (10^9^–10^5^ mol/L) for 1 day resulted in estrogenic effects, evidenced by the upregulation of the estrogen‐responsive gene (Zhao et al. 2010). Another in vitro study indicated possible estrogen‐mimicking effects of BF after exposing JEG‐3 cells to the pyrethroid for 1–4 days (Zhao et al. 2014). BF suppressed the 17β‐HSD‐17 beta‐hydroxysteroid dehydrogenase that catalyzes the interconversion of estrone to estradiol. This may result in low estradiol and possibly sexual dysfunction. The endocrine system communicates normally with other biological entities, including the reproductive system. Four included studies on the reproductive effects of BF were identified. The endocrine and reproductive communications during pregnancy depend on the hormonal integrity of the maternal‐fetal system. This system is bridged yet demarcated by a fetal‐placental core of trophoblast cells expressing estrogen receptors (Hemberger and Dean 2023). Exposure of JEG‐3 cells to BF has been reported to elicit endocrine perturbations in the trophoblast cells (Zhao et al. 2014).

The USEPA conducted several studies to determine the endocrine disruptor status of BF using the WoE approach (Table S12). The USEPA Endocrine Screening Program utilizes in vitro and in vivo screening assays to identify activity against several potential receptors that may indicate potential endocrine activity (USEPA 2015). The data were assessed using several assays designed to study interaction defined by agonism and antagonism at the estrogen and androgen receptors, altered steroidogenesis, as well as hypothalamic–pituitary‐gonadal and hypothalamic–pituitary‐thyroid perturbations. Based on WoE analysis, many of the USEPA Endocrine Screening Program assessment results of BF were negative in tier 1 assays for potential interaction with estrogenic, androgenic, and thyroid pathways, with no significant effects observed in vivo (Table S6). The only notable estrogen‐related effect in mammalian studies was a slight decrease in ovarian weight, while avian studies indicated cracked eggs without other reproductive changes. In the male pubertal rat and FSTRA studies, no androgen‐specific effects were observed in the absence of overt toxicity. There was no evidence of thyroid interaction; minor findings of thyroid gland atrophy in an amphibian assay did not indicate developmental delays. Overall, no treatment‐related thyroid effects were noted in the pubertal rat studies. The USEPA concluded that BF demonstrates no endocrine toxicity based on these data.

In the female reproductive system, in vitro evidence from included studies showed an alteration of luteinizing hormone/human chorionic gonadotropin‐induced ovulatory genes (Liu et al. 2011a). In vitro assessment of the reproductive function of epididymal spermatozoa exposed to BF 0.1, 1, 10, and 100 μM for 90 mins showed a decrease in sperm viability and motility (Bae and Kwon 2021; Bae et al. 2024). Ham et al. (2020) reported cell arrest and induction of apoptosis, leading to a decrease in the overall proliferation of Sertoli and Leydig cells. However, based on WoE evaluation of guideline studies, USEPA and EFSA concluded that BF is not toxic to the reproductive system (Table S13). A two‐generation reproduction dietary study conducted in rats did not indicate increased juvenile sensitivity to BF, and it determined a NOAEL of 3.0 and 5.0 mg/kg per day in males and females, respectively. The study only recorded the female LOAEL, which was 5.0 mg/kg per day (USEPA 2020). Tremors were noted only in these females of both generations, with one parental generation rat observed to have clonic convulsions and no observed effects in the offspring. Offspring toxicity assessment had a NOAEL of 5.0 mg/kg per day (No LOAEL was observed). In another study in which rats were orally treated with BF, maternal toxicity was observed with NOAEL and LOAEL of 0.88 and 1.77 mg/kg per day, respectively, based on tremors during gestation. Following a similar assessment in 1984, maternal toxicity was observed with NOAEL and LOAEL of 0.88 and 1.77 mg/kg per day, while developmental toxicity was observed with a NOAEL of 1.77 mg/kg per day (LOAEL was not observed). In the same assessment in 2001, BF had NOAEL and LOAEL of 7.1 and 15.5 mg/kg per day, respectively, in the mothers. Developmental toxicity was observed with a NOAEL of 15.5 mg/kg per day, respectively (LOAEL was not observed). In a rabbit study, maternal toxicity was observed with NOAEL and LOAEL of 2.36 and 3.5 mg/kg per day. In the developmental toxicity assessment, BF had a NOAEL ≥ 7 mg/kg per day (LOAEL was not observed). ECHA reported a NOAEL of 2.7 mg/kg per day for maternal toxicity, teratogenicity, and embryotoxicity following a post‐mating exposure of rabbits to 2.67, 4.0, and 8.0 mg/kg per day (Table S13).

#### 3.4.5 Other Effects

Oxidative status, enzyme activities, and pro‐inflammatory changes are measurable indicators of liver and kidney functions. While the liver is the primary epicenter of BF toxic effects and plays a crucial role in metabolism and detoxification (Treinen‐Moslen 2001), the kidney is vital in excreting unwanted metabolic products. We identified five studies that demonstrated hepatic and renal changes induced by BF. One study used the pyrethroid as a model of renal injury by exposing adult albino rats to BF (7 mg/kg per day) for 30 days (Abdel‐Wahhab, Elqattan, et al. 2024). Changes in oxidative status were characterized by decreased antioxidant markers (Abdel‐Wahhab, Elqattan, et al. 2024; Dar et al. 2019; Zhang et al. 2015). Oral BF (1.7 and 5.1 mg/kg for 5 days) effects on hepatic cytochrome p450 (CYP) enzymes were characterized by induction of CYP1A (Abdou et al. 2010). Liver function biomarkers were also decreased following 7 and 28 days of exposure to the pyrethroid in rodents (Pylak‐Piwko and Nieradko‐Iwanicka 2021; Zhang et al. 2015). Increased pro‐inflammatory cytokines IL‐1β, TNF‐α, and IFN‐γ were associated with BF exposure (1.5–8.5 mg/kg up to 30 days), renal and hepatic injury (Abdel‐Wahhab, Elqattan, et al. 2024; Pylak‐Piwko and Nieradko‐Iwanicka 2021).

Studies regarding metabolic, cardiovascular, respiratory, dermal, and ocular effects in humans and animals are still scarce. Three studies (1 animal, 1 in vitro, and 1 human study) showed BF‐induced metabolic changes (Liang et al. 2022; Wei et al. 2019; Xiang et al. 2018). Orally administered BF (0.6 mg/kg per 14 days for 6 weeks) elicited fat accumulation in C57BL/6 female mice because of increased fatty acid uptake and inhibition of lipolysis (Wei et al. 2019). Although this dose is lower than the NOAEL from the rodent study (Lewis et al. 2016), its lipid‐altering effect at this relatively safe dose may indicate a potential health risk. The in vitro approach using human hepatoma cells incubated with BF (10^−9^–10^−5^ M) for 2 h demonstrated BF‐induced upregulation of lipogenic genes and inhibition of ꞵ‐oxidation (Xiang et al. 2018). Proatherogenic effects, characterized by an increase in total cholesterol, LDL‐cholesterol, native LDL‐apoB‐100, and oxidized LDL, were recorded in adult male Wistar rats exposed to BF (3 mg/kg) for 90 days (Feriani et al. 2018).

Glucose dysregulation was assessed using the homeostasis model assessment of insulin resistance (HOMA‐IR), fasting plasma glucose (FPG), impaired fasting glucose (IFG), fasting plasma insulin (FPI), and abnormal glucose regulation (AGR). An evaluation of BF's effects on glucose homeostasis in the Chinese population (*n* = 3822) suggests that the pyrethroid is associated with glucose dysregulation (Liang et al. 2022). Although there was no positive association between BF and the risk of Type 2 diabetes mellitus (*p* > 0.05), the pyrethroid elevated the odds ratios of IFG and AGR (*p* < 0.05). Participants with BF exposure showed a 0.40 mmol/L, 11.07%, and 22.50% increase in FPG, FPI, and HOMA‐IR, as well as a 119.97% and 48.88% increase in IFG and AGR, respectively (*p* < 0.05). Although a few human case studies have recorded respiratory irritation and chest pain, the pyrethroid has yet to meet the EU classification criteria for this endpoint. Based on dermal and ocular toxicity studies (Table S9), BF is considered non‐irritating in rodents according to regulatory assessments of dermal and ocular effects (ECHA 2009; FAO 2009; USEPA 2020). Altogether, multiple homeostatic dysregulations of endocrine, reproductive, and metabolic communication are consistently associated with BF's biological effects.

## 4 Discussion

The dearth of systematic reviews that integrate regulatory assessments of BF toxicology and evaluate risk using a WoE approach highlights a significant knowledge gap, reflecting the limited scope of preclinical and clinical investigations. The current review examined studies reporting BF disposition and biological effects from health and regulatory perspectives. Findings from the evaluated studies support the hypothesis that BF interferes with biological systems, the safety implications of which must be examined through hazard identification and characterization, dose–response evaluation, and exposure risk assessment.

Our review highlights the importance of animal and in vitro research in identifying hazards, given the paucity of relevant human studies on its toxicity. Hazard is an agent's intrinsic or inherent ability to cause harm (McCarty et al. 2020). BF's potential to cause harm is dependent on its disposition. Approximately 14% of studies indicate that the lipophilicity of BF is associated with rapid toxicokinetics. BF's rapid distribution and elimination suggest a low potential for bioaccumulation. Higher doses of BF may have a longer elimination half‐life, regardless of the administration route. Although the basis is unclear, it suggests that dose may not be the sole factor influencing BF disposition (Gammon et al. 2012, 2014; Hughes et al. 2016). These measures of disposition, which rely on exposure levels, are crucial in evaluating BF's hazard potential and may provide information on regulatory stringency (Barlow et al. 205). Comparative data on local environmental concentrations and human body burden levels (low, moderate, and high) are essential to understand the varying regulatory strictness across countries and regions. Detection of BF in human samples confirms systemic exposure, with reports from parts of Asia and South America indicating higher potential body burdens (Abd Al‐Zahra and Ahmed 2018; Anand et al. 2021; Corcellas et al. 2012; Liang et al. 2022; Yoshida et al. 2021). However, the limited and uneven monitoring data continue to restrict robust regional comparisons.

Many studies indicate potentially harmful effects of BF on cells, though mutagenic studies were largely scarce. Although one study demonstrated DNA damage using the comet assay (a gel electrophoresis‐based method), it lacked evidence for gene mutation and was not validated in vivo (Bouaziz et al. 2020). WoE analysis considers gene/chromosomal aberration with in vivo validation more significant than primary DNA damage (USEPA 2008). As such, the single study is inadequate to assess BF's mutagenic potential. Moreover, DNA damage in the comet assay has a short lifespan and poses difficulty in distinguishing its source. Thus, this potential limitation, coupled with the paucity of mutagenic studies, limits adequate assessment of mutagenic risk based on peer‐reviewed studies.

The overall guideline genotoxicity data assesses gene mutation in bacterial and mammalian cells (in vitro), clastogenicity (in vitro and in vivo), and aneugenicity (in vivo) (EFSA 2023). These non‐threshold endpoints require multiple lines of evidence from various in vitro and in vivo assays for adequate mutagenic risk assessment. Generally, most guideline studies tested negative for the mutagenic potential of BF. Tumors observed in guideline studies are considered weak and irrelevant to humans (FAO 2009; USEPA 2020). The EFSA raises reliability concerns on gene mutation tests, including the sister chromatid exchange test (TG 479), in vitro unscheduled DNA synthesis assay (TG 482), and in vivo sex‐linked recessive lethal assay in *Drosophila melanogaster* (TG 477). Due to the low number of cells analyzed, precipitation at all dose levels, and exposure conditions, these tests deviated from current standards, leading to their replacement by the OECD in April 2014. In addition, the in vivo micronucleus studies generated reliability concerns by EFSA due to unproven bone marrow exposure using lethargy and closure of the eyes. These tests fall short of current standards, as these clinical signs do not reflect bone marrow exposure characteristics of the BF (pyrethroid) class and plasma analysis. EFSA, therefore, determined that BF's genotoxicity cannot be assessed due to outdated OECD tests and recommended that existing toxicological reference values be withheld (EFSA 2023).

In examining immunotoxic effects from a regulatory perspective, the USEPA found no convincing evidence of immunotoxic effects of pyrethroids based on functional immunotoxic assessment and does not require further immunological testing. In 2013, the Hazard and Science Policy Council recommended waiving immunotoxicity study requirements for BF, citing the goal of reducing laboratory animal use while upholding scientific rigor in pesticide evaluations (USEPA 2020). The European Union includes functional immunotoxicity testing assessment of the immune system in its 90‐day rodent study, not as a core requirement. Immunotoxic changes are susceptible to stress and less sensitive to systemic toxicity endpoints. Thus, immunotoxic effects reported in included studies may be attributed to secondary effects of stress or systemic toxicity. As such, they present no convincing risk to the immune system. While data on immunotoxic and inflammatory effects provide potential mechanisms for future risk assessment, further studies are necessary to determine if immune changes are primary or secondary effects.

Neurotoxicity is the critical effect used by the USEPA and EFSA to establish the toxicological values of BF. Literature and guideline studies demonstrated an association between neurotoxicity and BF exposure across several animal strains. In line with guideline studies, behavioral and motor impairment associated with anxiety, depression, and Parkinson's disease was consistent across most studies, lending weight to the association between BF and neurotoxicity. However, limitations in the study design, such as using a single dose group, narrow dose spacing, and reliance on a single model‐based behavioral approach, may constrain the comprehensive risk assessment (Abdel‐Wahhab, Sayed, et al. 2024; Gargouri, Yousif, Attaai, et al. 2018; Gargouri, Bhatia, et al. 2018; Gomaa et al. 2021; Holton et al. 1997; Syed et al. 2018, Syed et al. 2016; Zhang and Zhang 2024).

Endocrine risk assessment requires a WoE approach that considers evidence from multiple biological levels of organization (USEPA 2015). This approach is integral to the USEPA Endocrine Screening Program evaluations, which include (i) evaluating the quality and relevance of individual studies for potential endocrine interactions, (ii) integrating data across levels of biological organization, examining complementarity and redundancy in responses, (iii) characterizing the primary evidence and conclusions, and (iv) determining if additional testing is needed based on the evidence and conclusions (USEPA 2020). In vitro studies, which comprised over 80% of the included endocrine studies in the literature, demonstrated positive findings regarding estrogenic receptor activity and gene expression (Yang et al. 2022; Zhang et al. 2016, 2018; Zhao et al. 2010, 2014). The preponderance of in vitro evidence is undermined by the lack of sufficient supporting in vivo data to establish clear endocrine effects. Although an in vivo study noted gene changes related to testosterone synthesis, these were associated with changes in liver weight, suggesting systemic effects (Jin et al. 2014). Blinding and poor exposure characterization constituted potential sources of bias in these studies. Overall, the insufficient evidence, combined with performance and detection bias, makes the literature findings unconvincing regarding endocrine effects. Notwithstanding, the USEPA's assessments showed that BF was negative in Tier 1 in vitro assays for estrogen, androgen, and thyroid hormones. While potential estrogenic/antiestrogenic activity was reported in some non‐guideline in vitro assays, the in vivo assays did not support these positive responses.

Evaluation of reproductive effects included a decrease in viability and motility of male germ cells (Bae and Kwon 2021; Bae et al. 2024), endocrine changes in trophoblast cells (Zhao et al. 2014), alteration in ovulatory genes (Liu et al. 2011a) and cell arrest and induction of apoptosis with an overall decrease in the proliferation of Sertoli and Leydig cells (Ham et al. 2020). These were all limited to one level of biological organization—cells. While these events provide mechanistic data on the cellular effects of the pyrethroid, they show no convincing evidence of reproductive effects across other levels of biological organization. The multigeneration guideline studies provide sufficient evidence indicating that BF is not a reproductive hazard. Although ECHA suspected fetotoxicity in rabbits based on abortions and early delivery observed at mid and high doses, these were not relevant, as they were attributed to *Pasteurella multocida* infection (ECHA 2009). The significant changes in ovary weights of F1 but not in F2 generations, live birth index, and incidence of stillborn pups observed in the rat multigeneration study by ECHA were not dose‐related. Although guideline‐based data indicate no teratogenicity or fetal toxicity for BF, potential transgenerational impacts cannot be excluded. Current LOAEL and NOAEL derived benchmarks rely on relatively less sensitive endpoints (e.g., mortality and body weight), potentially overlooking more sensitive indicators, such as epigenetic modifications (e.g., DNA methylation shifts, histone modifications, and altered expression of non‐coding RNAs). Real‐world exposures, e.g., BF residues in infant formula (Galindo et al. 2024), and evidence from other pyrethroids showing early‐life epigenetic alterations (Vester et al. 2020; Bordoni et al. 2019; Xia et al. 2013), support the need for more sensitive evaluation. Environmentally relevant BF levels have been predicted to affect population sustainability through epigenetic changes (Brander et al. 2022). As regulatory multigenerational studies are limited to a 2‐year duration using relatively less sensitive endpoints, extended studies integrating molecular (epigenomic) with standard reproductive toxicity measures are recommended to establish BF's safety benchmarks.

From the foregoing, hazard characterization reveals neurotoxicity, with limited evidence for mutagenic, immunotoxic, endocrine, and reproductive effects. Since neurotoxic endpoints are the critical effects for setting BF toxicological benchmarks, relevant pathways establishing their causality are crucial for risk assessment and regulatory decision‐making (Guyton et al. 2018; Keller et al. 2012; Wikoff et al. 2019). They require the integration of key events, providing strong evidence for the causality and plausibility of BF's neurotoxic hazard (Meek and Wikoff 2023). Electrophysiological data suggest that BF alters voltage‐gated channels by affecting burst rates, calcium signaling, Na^+^/K^+^, and Mg^2+^ ATPase activities (Cao et al. 2012; Gargouri, Yousif, Bouchard, et al. 2018; Gargouri et al. 2019; Mohana and Prakhya 2016). Nonetheless, these events were not quantitatively assessed in the literature. Although other potential mechanisms like oxidative stress, apoptosis, and protein/gene expression were largely highlighted, these biochemical events indirectly measure neuronal damage and represent multiple endpoints (Deepika et al. 2020; Meek and Wikoff 2023). However, a strong quantitative assessment of these events could further boost the causal evidence of BF's neurotoxic characterization.

Robust dose–response data must corroborate the identified hazard to adequately assess the risk. This element of risk assessment is a prerequisite for selecting points of departure and minimizing their uncertainties throughout the process (USEPA 2020). Most guideline studies used the NOAEL and LOAEL as the primary points of departure to assess BF toxicity based on tremors. Typically, these studies employed only three dosing groups, differing in the tremor scoring metrics (USEPA 2020). The small dose spacing and potential variabilities in study design across these studies constitute potential weaknesses in these points of departure. Similarly, most included studies lack adequate dose selection, highlighting the limited understanding of dose–response relationships between BF and its biological effects.

The Wolansky study reveals robust dose–response data for extrapolating risk from BF exposure (Wolansky et al. 2006). Unlike the guideline studies, it utilized BMD instead of the traditional NOAEL‐based approach. BMD was based on the locomotor effect endpoint, measured at peak BF exposure levels. The locomotor effect provides a comprehensive dose–response relationship bearing on extensive dose selection (9 doses with 8–12 animals/group) and the use of a neurotoxin‐sensitive animal strain (Long Evans rat). The study, which the USEPA included, offers key advantages over NOAEL for risk assessment. Unlike the NOAEL approach, which does not fully account for uncertainties from study design, BMD incorporates the shape of the dose–response curve, quantifies uncertainty, and reduces dependence on parameters such as dosing volume and vehicle type. For example, Wolansky et al. (2007) reported a twofold decrease in the ED_50_ of BF when the corn oil dosing volume was reduced from 5 to 1 mL/kg. Besides its advantage in adopting the BMD method, it also contributes to a slight difference from the approach used by the European Union in deriving points of departure. Since this study, among others, was not available to the European Union peer review, the robustness of its reference values was deemed limited. This partly prompted recommendations for their withdrawal (EFSA 2023).

Based on current toxicological benchmarks, doses used in most studies were relatively higher than the acute (0.328 mg/kg per day) and chronic (0.013 mg/kg per day) population‐adjusted doses determined by the USEPA. Considering that the acceptable daily intake of BF is < 0.02 mg/kg per day, the high doses do not reflect real‐life exposure to the pyrethroid. Doses below safety benchmarks may not necessarily be risk‐free, despite their lower likelihood of adverse effects and regulatory concerns. Doses relevant to realistic human exposure are significant in risk assessment and must be determined. Given the paucity of epidemiological data, proper risk assessment requires extrapolating preclinical doses to human exposure. The EPA integrated the physiologically based pharmacokinetic (PBPK) model with in vitro and in vivo data to incorporate safety factors and extrapolate risk in accordance with the Food Quality Protection Act. While the PBPK model resolves dose and pharmacokinetic differences with significant predictive power in risk extrapolation, its complexities, transparency level, and insufficiency in estimating uncertainty factors for individual pyrethroid assessment constitute potential drawbacks. Due to existing limitations in the traditional risk assessment approach for BF and other pyrethroids, new approach methodologies for risk assessment may address these drawbacks. A proposed approach is the Next‐Generation Risk Assessment framework, which integrates toxicokinetics with new approach methodologies for toxicodynamics (Aznar‐Alemany and Eljarrat 2020; Elena et al. 2024). The novel framework not only surpasses the limitations of traditional risk assessment methods that rely on acceptable daily intake and default extrapolation models, but it can also assess the risk of combined and cumulative exposures (Fernandez‐Agudo and Tarazona 2025). This approach enables refined assessment of internal dose–response relationships, bioactivity thresholds, and cumulative mixture effects.

In addition, exposure to BF can occur through dietary (food and water), residential, occupational, and nonoccupational uses. The USEPA and EFSA hold different assumptions in the exposure risk assessment (Table S14). The USEPA's assessment evaluates the maximum application rate across all exposure scenarios. Risk was assessed as a series of acute exposures, assuming that repeated exposure does not decrease the points of departure (USEPA 2020). The US body deemed the BF residue chemistry database complete and set an enforcement limit of 0.05 ppm. The dietary exposure and risk assessments found no acute dietary risks for the US population. On the other hand, the EFSA assessed consumer risk based on both acute and chronic exposure, using two benchmarks: the acute reference dose and the acceptable daily intake, respectively. Based on benchmark scenarios from the EU and the FAO/WHO Joint Meeting on Pesticide Residues, the exposure assessments indicated a short‐term consumer health risk for strawberries (EFSA 2023). The European Commission requested a review of MRLs for BF by the EFSA. The EFSA raised concerns about the lack of validation data to monitor BF in spices, tea, and hops, and the need to review the Codex maximum limits for strawberries, mangoes, papayas, flowering and head brassica, kohlrabi, pulses, tea, and hops. It also recommended further risk management discussions to decide whether the existing EU MRLs for baby leaf crops, soya beans, muscle, and other edible offal from swine, bovine, sheep, goat, and equine should be maintained or lowered to the limit of quantification.

Non‐dietary exposure risks have been assessed for residential handling, post‐application (dermal or incidental), and occupational scenarios. The aggregate risk index (ARI) for residential handling exceeds the limit of concern, indicating no significant risk (Table S14). Post‐application dermal and/or incidental oral margins of exposures (MOEs) were of no risk concern following indoor treatments, treatments (shampoos) to dogs, and treatments to lawns/turf, except the maximum registered application rate for liquid formulations on lawns/turf (2.3 lb. ai/A). Occupational handler and post‐application risk estimates pose no risk concern for the existing uses of BF (MOEs ≥ 100 for dermal, ≥ 30 for inhalation, and ARIs ≥ 1). However, potential acute risks of concern have been identified for pollinators and other terrestrial invertebrates (USEPA 2020).

The USEPA has recommended addressing crop group conversions and commodity definitions, as well as harmonizing the United States and international tolerance levels. Precautionary labeling is advised to restrict access for vulnerable populations and protect pollinators (USEPA 2020). Conversely, the European Commission recently requested that EFSA conduct a stakeholder consultation on the reasoned opinions related to the targeted review of BF MRLs. The European authority concluded that some studies are potentially relevant to addressing data gaps and supporting the evaluation of toxicological reference values (EFSA 2023).

## 5 Conclusions and Perspectives

In this review, we evaluated the overall body of evidence based on quality and consistency. BF demonstrates several potential biological hazards. However, WoE analysis suggests that the mutagenic, immunotoxic, endocrine, and reproductive evidence for BF is largely unconvincing and inadequate for assessing risk. Poor study design, characterized by biases (Tables S7 and S8), small dose intervals, and reliance on a single model or line of evidence, undermines the quality of most literature studies and weakens their ability to assess risk. These shortcomings may, in part, account for the differences in the regulatory positions held by the USEPA and EFSA.

Most studies consistently observed oxidative stress, apoptosis, and other biochemical events linked to emerging evidence of anxiety‐like, depression‐like, and Parkinsonian‐like effects. Still, they lacked the quantitative dose–response data necessary to determine the causal relationship and human relevance of these findings. These biochemical events may constitute potential key characteristics and mechanistic constructs that establish causal relevance to neurobehavioral deficits. Quantitative assessment of their causal relevance requires well‐designed studies with stepwise documentation of key events and pathways, integrating biochemical, histological, molecular, electrophysiological, and behavioral endpoints across defined time courses and dose–response relationships. Whether BF's neurotoxicity may lead to quantitative neurodegenerative changes significant enough to cause major brain pathological findings depends on several modifiers, including BF dose, exposure duration, and species differences. These approaches not only provide quantitative evidence of neurodegenerative changes but may also address the limitation that these key characteristics overlap with toxicological domains beyond neurotoxicity, including carcinogenicity and immunotoxicity (Meek and Wikoff 2023).

Given the toxicological heterogeneity of pyrethroids, reliance on a single behavioral model offers limited insight into BF's neurobehavioral profile, as it captures only a narrow range of endpoints. Comprehensive risk assessment requires convergent evidence from multiple paradigms assessing distinct domains, including motor coordination (rotarod), spatial learning and memory (Morris water maze), recognition memory (novel object recognition), anxiety‐like behavior (open field, elevated plus maze) (Fajemiroye et al. 2014, 2016), and depression‐like behavior (forced swim, tail suspension). This multi‐model approach would provide a more complete and reliable characterization of BF's neurotoxicity for regulatory decision‐making.

Importantly, the relatively high doses above toxicological benchmarks, the paucity of human data, interspecies differences, and other factors may limit the conclusions of most studies on the pyrethroid's effect (Table 2). Much of the available traditional toxicology data for risk‐safety evaluations is mainly animal experimentation that identifies points of departure, such as NOAEL and LOAEL, from in vivo studies. Computational methods are under consideration to support systematic review efforts. Integrating new approach methodologies with traditional data for computational modeling can help predict complex adverse outcomes and substantiate information in the scientific literature. Further studies incorporating these approaches across several lines of evidence may substantiate available mechanistic data.

**TABLE 2:** Strengths and weaknesses of the literature on bifenthrin disposition and biological effects.

Bifenthrin outcomes	Strengths	Weaknesses
Bifenthrin disposition	(1) Studies examined oral, dermal, and inhalation routes. (2) Tissue concentration and time course of BF were reported.	(1) An in vitro study on dermal absorption may not reflect dermal absorption in humans. (2) Human studies are very scarce; available human studies did not analyse for confounding variables. (3) Peer‐reviewed studies on BF disposition besides those considered by regulatory bodies are limited.
Biological effects	(1) Cellular, neurotoxic, pro‐inflammatory, and oxidative effects were consistent in most studies. (2) Animal studies utilized multiple routes of administration, especially those that evaluated BF disposition. (3) Many in vitro studies directly assessed BF's biological effects through direct exposure of the pyrethroid to various human and animal cell lines. (4) A wide array of enzymes and molecular pathways was considered to assess BF's effect. (5) In endocrine assessments, in vitro studies used assays with high sensitivity and a proliferation endpoint sufficient to measure estrogenic action.	(1) Although highly sensitive assays were used for in vitro genotoxicity tests, robust data on the aneugenicity potential of BF in vivo are still lacking. (2) Neurobehavioral assessments of BF for depressive‐like and anxiogenic‐like effects by some studies only used a single model, for instance, a forced swimming test only for depressive‐like effects and an elevated plus maze only for anxiogenic effects. (3) Lack of blinding and poor exposure characterization: Most studies demonstrated no evidence of methods used to achieve randomization and concealment in study group allocation. (4) There is a lack of human studies; most human studies did not analyze confounding variables. (5) Most studies focused only on hazard characterization. (6) Interspecies differences may be an important limitation.

Finally, the USEPA deems BF's toxicological and risk assessment data robust and protective for approved uses. However, EFSA recommends withdrawing some existing MRLs and toxicological reference values due to concerns about their reliability. To address these discrepancies, we propose a full revisit and re‐evaluation of genotoxicity data per current scientific and regulatory standards of the OECD, a unified framework for detailed data sharing and accessibility, and an open policy dialogue between stakeholders in joint review panel meetings that promotes a collaborative strategy. These strategies may foster a consensus on the design and implementation of harmonized approaches that may close existing regulatory gaps.

## Conflicts of Interest

The authors declare no conflicts of interest.

## Supporting information


**Figure S1:** The main target of BF's toxic action is the cell membrane flux of NA^+^, Ca^2+^, Cl^−^, and K^+^, which may be related to symptoms of depression. Exposure to BF leads to oxidative stress.

**Table S1:** General physicochemical properties of bifenthrin.
**Table S2:** Regulatory status of bifenthrin.
**Table S3:** PECO statement.
**Table S4:** Inclusion and exclusion criteria.
**Table S5:** Summary of animal and in vitro studies on bifenthrin exposure.
**Table S6:** Summary of human studies on bifenthrin exposure.
**Table S7:** Risk of bias assessment for animal and in vitro studies on bifenthrin disposition and biological effects.
**Table S8:** Risk of bias assessment for human studies on BF disposition and biological effects.
**Table S9:** Summary of oral, dermal, inhalation, and eye toxicity studies.
**Table S10:** Mutagenicity in vitro tests.
**Table S11:**. Neurotoxicity studies.
**Table S12:** Endocrine studies.
**Table S13:** Reproductive studies.
**Table S14:** Non‐cancer exposure and risk assessment for registered uses of bifenthrin in the United States.

## Data Availability

Data is available in the article's Supporting Information.
