# Supplementary figures and images for: Bifenthrin Under Scrutiny: Revisiting Toxicological Evidence Amid Regulatory Gaps
Source: J Appl Toxicol. 2025 Sep 17;46(1):61–77. doi: 10.1002/jat.4929 (PMC12668871; doi:10.1002/jat.4929)

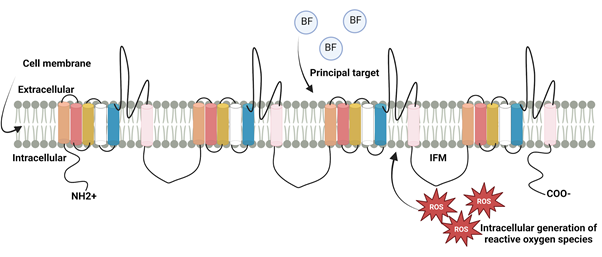

Supplement: Supplementary file 1 — Figure S1: The main target of BF's toxic action is the cell membrane flux of NA+, Ca2+, Cl−, and K+, which may be related to symptoms of depression. Exposure to BF leads to oxidative stress. [file JAT-46-61-s001.tif]
