# Supplementary material for: Bifenthrin Under Scrutiny: Revisiting Toxicological Evidence Amid Regulatory Gaps
Source: J Appl Toxicol. 2025 Sep 17;46(1):61–77. doi: 10.1002/jat.4929 (PMC12668871; doi:10.1002/jat.4929)
Supplement: Supplementary file 2 — Table S1: General physicochemical properties of bifenthrin. Table S2: Regulatory status of bifenthrin. Table S3: PECO statement. Table S4: Inclusion and exclusion criteria. Table S5: Summary of animal and in vitro studies on bifenthrin exposure. Table S6: Summary of human studies on bifenthrin exposure. Table S7: Risk of bias assessment for animal and in vitro studies on bifenthrin disposition and biological effects. Table S8: Risk of bias assessment for human studies on BF disposition and biological effects. Table S9: Summary of oral, dermal, inhalation, and eye toxicity studies. Table S10: Mutagenicity in vitro tests. Table S11:. Neurotoxicity studies. Table S12: Endocrine studies. Table S13: Reproductive studies. Table S14: Non‐cancer exposure and risk assessment for registered uses of bifenthrin in the United States. [file JAT-46-61-s002.docx]

**Bifenthrin under scrutiny: Revisiting toxicological evidence amid regulatory gaps**

Caroline VL Moreira^a^, John I Ogbu^a^, Kadja LC Monteiro^b^, Thiago M de Aquino^b^, Edeildo F da Silva-Júnior^c^, Alberto SS Filho^d^, Hamilton B Napolitano^d^, Christianah A Elusiyan^e^, Renê O do Couto^f^, Elson A Costa^a^ and James O Fajemiroye^ad*^

**Table S1.** General physicochemical properties of bifenthrin

| **IUPAC name** | (2-methyl-3-phenylphenyl) methyl (1S,3S)-3-[(Z)-2-chloro-3,3,3-trifluoroprop-1-enyl]- 2,2-dimethylcyclopropane-1-carboxylate |
| --- | --- |
| **Common name** | Bifenthrin |
| **Chemical formula** | C_23_H_22_ClF_3_O_2_ |
| **Molecular weight** | 422.868 g/mol |
| **Chemical name** | Cyclopropane carboxylic acid, 3-(2-chloro-3,3,3-trifluoro-1-propenyl)-2,2-dimethyl-, (2-methyl[1,1'-biphenyl]-3-yl) methyl ester, [1α,3α(Z)] |
| **Melting point** | 68 – 71°C |
| **Boiling point** | 453.2 °C at 760 mmHg |
| **Freezing point** | 51 -56 °C |
| **Density** | 1.212 kg l^-1^ |
| **Color** | Pale tan to off-white, waxy solid granules or viscous liquid with a faint, musty odor, and slightly sweet smell. |
| **Odor** | Weak aromatic odor, musty and slightly sweet smell |
| **Vapor pressure** | 1.81 x 10^-7^ mmHg at 25 °C |
| **Partition coefficient (Water/Octanol coefficient (Kow):** | 1 x 10^6^ |
| **Solvents** | methanol, ethanol, isopropanol, and cyclohexane |

Patty, 2001; Zang et al. (2020)

**Table S2:** Regulatory status of bifenthrin.

| **Processes** | **Regulatory background and status** | | | | |
| --- | --- | --- | --- | --- | --- |
|  | **FAO/WHO** | **EFSA** | **USEPA** | **CONTEXT** | **REFERENCES** |
| **Approval** | Not applicable | Approval in 2012 by Regulation (EU) No 582/2012.  Not currently approved.  An application for approval renewal was submitted in 2018 but rejected.  Approval expired in July 2019. | Approved.  First registered for use in 1985. | **FAO/WHO:** it only establishes and reviews specifications for BF to provide an international point of reference against which the material can be judged for regulatory purposes.  **EFSA:** BF is not approved for use as a plant protection product. Several concerns cited include the impossibility of assessing the potential contamination of groundwater by TFP acid; the possibility of risk underestimation to consumers due to the limited amount of available residue data; the lack of investigation on the metabolism pattern of the two isomers constituting BF; the risk to aquatic vertebrates has not shown to generate acceptable uses; the remaining uncertainty as regards the effects of the experienced bioaccumulation in fish of the active substance; and the high risks identified for mammals (long-term risk and secondary poisoning), earthworms (long-term risk), and non-target arthropods (in-field), in addition to the fact that the risk to non-target plants and non-target soil macro-organisms has not been sufficiently addressed.  However, is registered in Europe as a biocide for wood preservation.  **USEPA:** Registered for various crops, ornamentals, and turf. Most crops can be applied using ground, air blast, aerial, and chemigation equipment. Restricted entry interval (REI) for the proposed uses of BF is 12 hours. | FAO, 2009; USEPA, 2020; EFSA, 2020 |
| **MRL review and approval** | Approved | Reviewed | Reviewed | **FAO/WHO:** Currently approves CODEX MRLs.  **EFSA:** MRL application Art. 12 conﬁrmatory data (citrus fruits, strawberries, cane fruits and animal commodities) and import tolerance in sweet corn and maize grain. Not yet legally implemented.  Implementation of certain CXLs adopted by CAC 2011 following assessment by EFSA (EFSA, 2011) and discussion in CCPR 43(2011) (i.e., CXLs for cane fruits, tomato, pepper, aubergine, cotton seeds, products of animal origin from swine, bovine, sheep, and goat). Legally implemented by Regulation (EU) No441/2012.  Implementation of CXLs adopted by CAC 2016 following assessment by EFSA (EFSA, 2015) and discussion in CCPR 48(2016) (i.e., CXL on blueberries, grapes, peas with and without pods). Legally implemented by Regulation (EU) 2018/687.  **USEPA**: considers international MRLs established by the Codex but may establish a tolerance that is different from that of Codex due to the absence of the commission’s MRLs for some fruits. | FAO, 2009; EFSA, 2023; USEPA 2020. |
| **Toxicity classification and conclusion** | BF is neurotoxic.  BF is unlikely to be genotoxic and teratogenic. | Carc. 2, H351 ‘suspected of causing cancer’.  Acute Tox 3, H331 ‘toxic if inhaled’.  Acute Tox 2, H300 ‘fatal if swallowed’; STOT RE 1, H372 (nervous system) ‘causes damage to organs’.  Skin Sens. 1B, H317 ‘may cause an allergic skin reaction’, but BF does not fall under the cut-off criteria. | Toxicity category I by the acute oral.  Toxicity Category III by the acute inhalation route of exposure.  Toxicity Category III by the acute dermal route of exposure.  Toxicity Category III for acute eye irritation potential and Toxicity.  Toxicity Category IV for skin irritation potential.  “Class C” carcinogen, i.e., ‘Possible human carcinogen’ | **EFSA:** Classifications were based on CLP criteria after evaluation of results from toxicity studies.  **USEPA**: Oral, inhalation, dermal, and ocular are classified based on their LD_50_ and LC_50_ (inhalation).  Carcinogenicity studies based on a mouse study in which the high-dose males showed an increased incidence of urinary bladder tumors. | FAO, 2009; EFSA, 2023; USEPA 2020 |
| **Endocrine effects** | - | Endocrine disruptor assessment has not been performed according to ECHA and EFSA guidance (ECHA and EFSA, 2018), and scientiﬁc criteria (EC, 2018) have not been performed (EFSA, 2023). | No convincing evidence of potential interaction with estrogen, androgen, or thyroid pathways. | USEPA utilized a weight-of-evidence approach, which integrated various factors to conclude the endocrine effects. | USEPA 2020 |

MRL: Maximum residue limit; Codex: Codex Alimentarius Commission; CLP: Classification, Labelling and Packaging; EDSP: Endocrine Disrupting Screener Program; ECHA: European Chemical Agency; TFP: (cis,trans)-4-(2-Chloro-3,3,3-trifluoro-1-propenyl)-3,3-dimethylcyclopropanecarboxylic acid.

**Table S3.** PECO statement

|  | **Human evidence** | **Animal evidence** | ***In vitro* evidence** |
| --- | --- | --- | --- |
| **Population** | Human populations without restrictions. | Rodents | *In vitro* models using organs, tissues, cell lines, or cellular materials. |
| **Exposure** | BF and metabolites measured *in vivo.* | BF is administered at varying doses, lengths, and routes. | BF is administered at varying doses and lengths. |
| **Comparator** | A comparison population exposed to lower levels (or no exposure) of BF; Experimental studies should include unexposed or controlled population. | Animals with no exposure to BF (control). | Experimental studies should include untreated or control group. |
| **Outcomes** | BF disposition and biological effects. | BF disposition and biological effects. | BF disposition and biological effects. |

**Table S4:** Inclusion and exclusion criteria

| **PECO** | **Inclusion** | **Exclusion** |
| --- | --- | --- |
| **Population** | Human, rodent, and *in vitro* models. | All other models. |
| **Exposure** | Exposure to BF and/or its metabolites. | Exposure not described or determined. |
| **Comparator** | Must include vehicle or untreated group; low or no BF levels in the case of studies on BF disposition. | Articles lacking appropriate groups that allow for comparison. |
| **Outcome** | Articles that evaluated BF’s disposition, biological effects, toxicity, and mechanism of action. | Articles that focused extensively on chemistry without evaluating the mechanism of action, biological effects on all aquatic species (outside population of interest), soil, and its organisms. |
| **Other parameters** | | |
| **Language** | **English** | **Any other language** |
| **Duplicate articles** | Selected automatically and manually using Zotero. | Studies on BF related to plant species and work that only contained the abstract. |
| **Study types** | Original articles. | Reviews, book chapters, posters, and contents. |

**Table S5.** Summary of animal and *in vitro* studies on bifenthrin exposure

| **Biological outcome** | **Population** | **Exposure measure and duration** | **Comparator** | **Biological markers and parameters** | **Main findings** | **Author(s)** |
| --- | --- | --- | --- | --- | --- | --- |
| **Renal effects** | Adult male albino rats | BF (7 mg/kg/day) for 30 days. | Control | Serum values of urea, creatinine, ALAT, and ASAT.  Renal inflammatory (IL‑1β, TNF‑α & IFN‑γ).  Caspase‑3.  Oxidative stress (MDA and NO). | Increased ALAT, ASAT, IL‑1β, TNF‑α & IFN‑γ, Caspase‑3; MDA and NO.  Decreased GSH, GPx, and SOD. | Abdel-Wahhab et al. (2024b) |
| **Hepatic effects** | Male and female BALB/c mice. | BF (2, 4, and 8 mg/kg) for 7 days. | Control | Serum ALT, AST and ALP.  LDH, SOD, and MDA. | ALT and AST elevated by 8 mg/kg group.  Significantly altered MDA.  Increased mitochondrial ROS generation. | Abdou et al. (2010); Zhang et al. (2015) |
|  | Sprague Dawley rats for 5 days. | BF (1.7 and 5.1 mg/kg) for 5 days. |  | Liver CYPs | Induced of CYP1A |  |

| **Reproductive effects** | | Immature female Sprague–Dawley rats. | | BF 0.5 mg/kg and 5 mg/kg, IP for 5 days. | | Control | | Profiles of LH/hCG-induced ovulatory genes in rat ovaries *(in vivo*) and granulosa cells (*in vitro*).  LH/hCG-induced PGE2 production. | | Altered expression profiles of LH/hCG-induced ovulatory genes in ovaries and granulosa cells.  Inhibited LH/hCG-induced PGE2 production in ovarian granulosa cells. | | Liu et al. (2011a); Bae et al. (2024); Bae and Kwon, (2021); Ham et al. (2020) | |
| --- | --- | --- | --- | --- | --- | --- | --- | --- | --- | --- | --- | --- | --- |
|  |  | Sprague-Dawley rat granulosa cells | | BF (1 nM, 10 nM, 100 nM and 1000 nM) for 30 mins. | |  |  |  |  |  |  |  |  |
|  |  | *In vitro* Epididymal spermatozoa collected from mice Sperm cells and mature ICR male and female mice. | | BF (0.1, 1, 10, and 100 μM) for 90 mins. | |  |  | Sperm viability and motility; Spontaneous acrosome reaction and capacitation status.  Expression of DEPs.  Intracellular ATP levels.  Levels of phospho-PKA and phosphor-tyrosine substrate. | | Decreased sperm viability and motility, spontaneous acrosome reaction, and capacitation.  Decreased expression of DEPs.  Blastocyst formation, intracellular ATP levels, phospho-tyrosine phosphate.  Increased phospho-PKA levels. | |  |  |
|  |  | *In vitro* Immature mouse Leydig (TM3) and Sertoli (TM4) cells. | | BF (0, 0.2, 0.5, 1, 2, and 5 mM) for 24h. | |  |  | Proliferation and viability of mouse TM3 and TM4 cells.  Cell cycle progression; mitochondrial function and intracellular Ca levels. | | 52% (0µm) and 68% (5µm) decrease in proliferation, Induced cell cycle arrest and apoptosis.  Decreased mitochondrial function and increased intracellular Ca levels.  Downregulates the expression of the genes related to cell cycle progression and testis functions. | |  |  |
| **Cellular effects** | | *In vitro* (Cultured neuronal cells) assessed using Microelectrode arrays | | BF (0.01, 0.1, 1, 10, and 100 μM) for 30 mins. | | Control | | Microelectrode arrays parameters MFR, mean MBR, and number of nAC. | | Increased MFR, MBR.  Increased the average nAC at lower concentrations before decreasing at higher concentrations. | | Cao et al. (2014); Beghoul et al. (2017) |  |
| **Cellular effects** | | Female *Wistar* rats | | BF (2.6 mg/kg) for 6 months. | | Control | | Brain mitochondria MDA, GSH, CAT, and GST levels.  Brain mitochondria swelling. | | Increased MDA but not GSH and decreased CAT.  Increased mitochondrial swelling. | | Baskar and Murthy, (2018); Bouaziz et al. (2020); Liu et al. (2008); Liu et al. (2009); |  |
|  |  | *In vitro* human colorectal (HCT)-116 cells. | | BF (5 to 200 µM) for 24h. | |  |  | Cell viability, ROS species, MDA levels, DNA fragmentation, mitochondrial membrane potential, caspase, and MAPK activation. | | Decrease percentage cell viability and mitochondrial potential, increased ROS production, MDA levels, DNA fragmentation, caspase, and MAPK activation. | |  |  |
|  |  | *In vitro* Primary mouse cortical neurons from C57Bl/6J mouse pups. | | Nanomolar BF (0.1 µM (acute) for 50 mins (0.01–1.0 µM) for chronic. | |  |  | Frequency and amplitude of synchronous Ca^2+^ oscillations (SCOs). | | Increased frequency of SCOs and decreased amplitude of SCOs.  Neither altered membrane potential or voltage-gated currents. | |  |  |
|  |  | *In vitro* Hep G2 cells. | | cis-BF at 10 or 20mg/L for 6h and cis-BF (0, 5, 10, 20, and 40 mg L−1) for 12h. | |  |  | Cell viability, ROS, apoptosis. | | 6h treatment: 1S-cis-BF Decreased cell viability, also induced ROS production, up-regulated Bax protein expression, and down-regulated Bcl-2 expression levels.  12h treatment: Dose-dependent reduced cell viability; 1S-cis-BF was more apoptotic than 1R-cis-BF; 1S-cis-BF and cis-BF resulted in increased levels of the phosphorylated forms of JNK/MAPKs. | |  |  |
|  |  | *In vitro* FL cells (human amniotic cells). | | BF (0, 25, 5, 10, and 20 mg L−1) for 4h for oxidative assessment.  BF (5 and 50 mg L−1) for apoptosis assessment. | |  |  | Cell viability, ROS production; DNA damage indicated by tail lengths. | | Decreasing order of toxicity to FL cells was 1S-cis-BF (55%) < racemate (60%) < 1R-cis-BF (82%).  1R-cis-BF increased ROS.  Significant differences in tail length; loss of nucleolus and cytoskeletal integrity with 1S-cis-BF. | |  |  |
| **Cellular effects** | | *In vitro* PC12 cells. | | Racemate, and each enantiomer BF (1, 5, and 25µm) for 25 h.  Racemate and each enantiomer of cis-BF (10^-9^ mol L^-1^ to 5 × 10^-25^ mol L^-1^) for 24 h. (except for the ROS assay, which needed 6 h).  BF (10^–13^, 10^–10^, 10^–7^, 10^–6^, 10^–5^ and 10^–4^ M) for 72 h. | | Control | | Expression levels of HSP90, HSP70, and HSP60 mRNA.  Cu–ZnSOD, MnSOD, GST and CAT mRNA levels.  Cell viability, release of extracellular LDH; SOD, MDA. | | Increased expression of HSP90, HSP70, and HSP60 mRNA by cis-BF.  Increased MDA and decreased SOD.  Upregulation of Cu–ZnSOD, MnSOD, GST and CAT mRNA levels by 25µm cis-BF.  Slight reduction in cell viability at doses up to 10^-6^ mol L^-1^;  Elevation in LDH release by cis-BF and its enantiomers.  Reduced cell viability at 10^–5^ M and 10^–4^ M.  Inhibited neurite outgrowth. | | Lu et al. (2011); Lu, (2013); Yang and Li, (2015); Mohana and Prakhya, (2016); Mukhtar et al. (2022); Tran et al. (2006) | |
|  |  | *In vitro* Cortical neurons. | | BF (10 and 50 µm). | |  |  | Electrophysiological parameters. | | Caused a pronounced late current that persisted at the end of a depolarizing pulse, a slowly decaying tail current following repolarization, and significant resting modification.  Caused a concentration-dependent hyperpolarizing shift in steady-state activation and inactivation as well as slowed recovery from channel inactivation. | |  |  |
| **Oxidative effects** | | *In vitro* Human erythrocyres. | | BF (0, 42.2, 211, 1055 ppm) for 4 h. | | Control | | MDA concentrations, CAT and SOD. | | Enhanced lipid peroxidation and decreased antioxidative enzyme activities. | | Sadowska-Woda et al. (2010); Dar et al. (2013); Dar et al. (2015a); Dar et al. (2019) Tatipamula and Kukavica, (2022) | |
|  |  | *In vitro R*ats erythrocytes from Fourteen healthy albino rats of either sex. | | BF 1 ppm for 3h. | |  |  | SOD, CAT, GST, and MDA. | | Decreased SOD, CAT.  Increased MDA and GST. | |  |  |
|  |  | Rats | | BF (5.8 mg/kg, 2 groups) for 20 or 30 days  BF (5 mg/ml, 2 groups) and (45 mg/Kg dermally, 2 groups) for 20 or 30 days. | |  |  | Blood MDA, GSH, SOD, CAT, GPx.  Brain and erythrocyte acetylcholinesterase (AChE).  GPx of the kidney, lung and liver. | | Increased MDA; Decreased GSH, CAT, SOD and GPx.  5mg/ml:Decreased AChE.  45mg/Kg: Non-significant (p>0.05) decrease of cholinesterase in plasma and erythrocyte lysate. | |  |  |
| **Cardiovascu lar effects** | | Adult male *Wistar* rats | | BF (3 mg/kg) for 90 days. | | Control | | Blood and aorta Total cholesterol (TC), Low-density lipoproteins (LDL-cholesterol), LDL-apo B-100, and ox-LDL.  LDL receptor level.  Pro-inflammatory cytokines IL-2, IL-6, TNF-α. | | Increased TC, LDL, LDL-apo B-100, and ox-LDL.  Decreased LDL receptor.  Increased IL-2, IL-6, TNF-α. | | Feriani et al. (2018) |  |
| **Neurotoxic effects** | | Male Parkin knock-out mice and C57BL/6 mice. | | BF 10 mg/kg bw/day (28 days). | | Control | | Behavioral parameters, Number of TH- positive cells in the substantia nigra. | | Decreased latency to fall, increased pole climbing time.  Decreased the TH‑Positive Cells in the Substantia Nigra.  Disrupted the Key Protein Expression of PD in the Substantia Nigra. | | Syed et al. (2016); Syed et al. (2018); Gargouri et al. (2018a); Gargouri et al. (2018b); Gargouri et al. (2018c); Gargouri et al. (2019) |  |
|  |  | Adult male albino rats | | BF 7mg/kg/day (30 days). | |  |  | Time of fall (for motor coordination), time spent in the quadrant (spatial memory).  Brain (Cortex, hippocampus and striatal) dopamine, serotonin, AChE-ase.  Brain MDA and NO, GSH, SOD, CAT and GPx.  Brain TNF-α and IL-1β. | | Decreased memory and motor performance.  Increased exploration, locomotion, and spatial learning impairments.  Brain dopamine, serotonin, AChE-ase.  Increased MDA and NO and decreased GSH, SOD, CAT and GPx. | |  |  |
|  |  | Male *Sprague-Dawley* rats | | BF (0, 50, 100 or 125 ppm (mg kg−1 diet) from gestation day 6 to lactation day 21. | |  |  | Acoustic startle response (ASR) amplitude.  Molecular modeling parameters. | | Tremors and clonic convulsions in dams and pups and slightly reduced ASR amplitude.  o-Me group may occupy a similar space to the 𝜶-CN group of cypermethrin. | |  |  |
| **Neurotoxic effects** | | Male Wistar rats | | BF (3.5 and 7 mg per kg body weight) once daily for 30 days. | | Control | | Behavioral parameters.  Levels of biogenic amines like DA and its metabolites, i.e. DOPAC and HVA, EPN, NE, and 5-HT in the cortex, corpus striatum, and hippocampus.  Protein carbonyl levels. | | Deficits in motor activity, motor incoordination, and cognitive impairment.  Altered levels of DA, DOPAC, HVA, EPN.  A decrease in the activity of acetylcholinesterase (AChE).  Increased protein carbonyl levels | | Gammon et al. (2019); Gomaa et al. (2021); Abdel-Wahhab et al. (2024a); Zhang and Zhang, (2024) |  |
|  |  | Adult male and female Wistar rats. | | BF at a dose level of 1/15 of LD_50_ daily in females throughout the gestation period.  LD_50_ used was determined from previous studies). | |  |  | Neonatal behavioral endpoints such as surface righting, pivoting, and negative geotaxis reflex.  Motor activity and coordination for adults.  CAT, SOD, and GPx | | Impaired pivoting in neonates.  Decreased locomotion in the open field and impaired rota-rod performance.  Reduced CAT, SOD, and GPx activity. | |  |  |
|  | | Adult male Long Evans rats. | | BF (1 and 5 ml/kg) | |  | | Neurotoxic endpoints. | | Increased fine tremor decreased motor activity and grip strength, and increased pawing, head shaking, click response, and body temperature. | |  | |
| **Immunotoxic effects** | | *In vitro*; human CD4+ H9, and Jurkat cell lines and the human promonocyte U937 cell line. | | BF (10^–4^, 10^–5^, 10^–6^, 10^–7^, 10^–8^, 10^–10^ and 10^–13^ M) for 0, 3, 6, 12, 24 and 48h. | | Control | | Cell viability; PHA.  Aggregate formation. | | Reduced cell viability at a concentration of 10^–4^ M by 100%.  BF did not inhibit PHA-induced cell aggregation in all cell lines tested.  BF to form aggregates stimulated homotypic aggregation in the H9 and Jurkat T-cell lines. | | Hoffman et al. (2006); Jin et al. (2012); Wang et al. (2017) |  |
|  |  | Younger and adult male mice (*Mus musculus*). | | 10 and 20 mg/kg of BF for 3 weeks. | |  |  | Body and immune organ weight; levels of Immune System-Related Gene Expression.  Serum and liver GSH and MDH.  Hepatic CAT and GPx. | | Decreased thymus weight in the adolescent but not adult mice; mRNA levels of TNF and IL2 in spleen but not liver.  Upregulation of thymus mRNA levels of IL2 and IL4.  Compared to serum, hepatic GSH content increased significantly in both the adolescent and adult mice exposed to 20 mg/kg BF.  Hepatic CAT and GPx activities were significantly altered, even in adolescent mice, after treatment with 10 mg/kg BF. | |  |  |
|  |  | C57BL/6 mice. | | BF (0, 5, 10, and 20 mg/kg/d) for 3 weeks. | |  |  | Spleen and thymus weights.  mRNA levels of the pro-inflammatory factors IL-1β, IL-6, CXCL-1, and TNF-α;  Splenocyte proliferation. | | Spleen and the thymus were inhibited in the cis-BF.  Inhibited mRNA levels of the pro-inflammatory factors IL-1b, IL-6, CXCL-1, and TNF-a, in peritoneal macrophages.  cis-BF inhibited splenocyte proliferation. | |  |  |
| **Endocrine effects** | | *In vitro* human adrenocortical carcinoma (H295R) cells. | | BF (0, 1, 10, 100 nM) for 24 h. | | Control | | Cell viability, basal cortisol and aldosterone production, steroidogenic genes involved in cortisol and aldosterone production.RHI | | No significant changes in cell viability.  Decreased basal production of cortisol and aldosterone.  Diminished the cAMP-induced cortisol production.  No significant changes in aldosterone level.  Significant down-regulation in basal mRNA levels of P450scc, 3βHSD, CYP17 and CYP11B1. | | Zhang et al. (2016); Zhang et al. (2018); Yang et al. (2022) |  |
|  |  | *In vitro* Chinese hamster ovary K1(CHO-K1) cells, rat hepatoma cells H4IIE and mouse 121 macrophage cells J774A and human HCC cell line. | | BF 10^-5^, 10^-6^ or 10^-10^ M for 24 h. | |  |  | Antagonistic effect on glucocorticoid (GC) activity.  Phosphoenol pyruvate carboxykinase expression  Mineralocorticoid (spironolactone) antagonistic activity. | | Significantly attenuated cortisol-induced GR transactivation and aldosterone-induced MR transcriptional activity at the highest tested noncytotoxic concentration  Inhibited cortisol induced PEPCK expression.  IC_20_=5.19 X10^-7^  IC_50_=1.44 X10^-6^  luciferase activity induced by aldosterone.  IC20=3.76 X10^-7^ and IC20=7.06 X10^-6^ | |  |  |
|  |  | JEG-3 cell line. | | Racemic and enantiomeric BF (10^−7^ to 10^−5^) for 1-4 days. | |  |  | Cell viability.  Progesterone and Human chorionic gonadotropin (hCG).  PR, 3β-HSD, 17β-HSD CYP17, CYP19, and HLA-G gene expression. | | Inhibited cell viability.  Racemic BF-induced progesterone and hCG secretion.  BF significantly induced PR, 3β-HSD, CYPs, and HLA-G gene expression in a dose-dependent manner.  BF downregulated 17β-HSD. | |  |  |
| **BF disposition** | | *In vitro* Rat liver microsomes (RLM) from 90-day-old Sprague-Dawley (SD) and 15-day-old SD rats.  Human liver microsomes (HLM). | | BF (0.52 mg of BF in 0.123 mL of 20% DMSO) for 60 mins. | | Control | | BF metabolites 4’-OH-BF and TFP acid.  Percent metabolism of BF.  Intrinsic clearance. | | TFP acid was also produced in microsomes without the co-factor NADPH.  4’-OH-BF produced with NADPH.  Metabolism of [14C]-BF; percent metabolism in human liver cytosol and microsomes was lower than that in the rat sub-cellular fractions.  BF undergoes metabolism primarily via NADPH dependent (oxidative) pathway. | | Shafer and Hughes, (2010); Scollon et al. (2011) |  |
|  |  | Male Long Evans rats | | BF (0, 0.1, 1, 2, 4, 6, 8, 12, 16 mg/kg) for 4 h or (0, 0.05, 0.5, 1, 3, 4.5, 6, 9 mg/kg) for 7 h. | |  |  | Blood and brain levels of BF (correlated with motor activity). | | BF exposure decreased motor activity from 20% to 70% in a dose-dependent manner at both time points.  The relationship between motor activity and brain concentration was not significantly different between the two-time points. | |  |  |
|  |  | In-vitro Primary cultures of neurons from Long-Evans rat pups. | | BF (0.05, 0.1, 0.5, 1.0, 5.0 and 10.0 µM) for 2.5, 5, 15, 30, 60 and 90 min. | |  |  | Concentration- and time-dependent accumulation of BF in neurons. | | Accumulation of all three pyrethroids was time- and concentration-dependent. | |  |  |
| **BF disposition** | | *In vitro* / Rat skin from male Long Evans rats and Dorsal skin obtained from adult male cadavers. | | BF (10, 20 and 100 nmol) for 24h. | | BF absorption levels. | | Percentage of BF dose in receptor fluid, skin wash, and skin both in human and rat skin for 24h. | | Receptor fluid: (rat skin=1-5%; human skin=1-2%).  Skin wash: (rat skin:53-71%; human skin=71-83%).  Dose remaining in skin (rat=26-43%; human=14-25%). | | Hughes and Edward, 2010; Gammon et al. (2014); Hughes et al. (2016) | |
|  |  | Male Long-Evans rats | | BF (0.3 or 3 mg/kg, p.o) for 0.25 h to 21 days and (0.3 mg/kg, 0.1 mL/kg, i.v) for 0.25 h to 21 days. | | Levels of BF in tissue. | | Blood and tissue concentration of BF. | | BF concentration 1–2-h post oral administration.  Blood=90 ng/mL Liver=1000 ng/mL.  Blood BF concentration decreased bi-exponential after i.v. | |  |  |
|  | | Male *Sprague-Dawley* rats | | BF (3.1 mg/kg orally, 0.018 mg/l inhalation for 2, 4, 6, 8 and 12h. | | Vehicle | | Maximum concentration of BF I brain and plasma, Area under the concentration versus time curve (AUC), terminal half-life. | | Increased BF concentrations in blood and brain. | |  |  |
| **Metabolic effects** | | C57BL/6 female mice. | | BF (0.6 mg/kg body weight) once every 2 days for 6 weeks. | | Control | | Body weight and serum TC.  Fat mass and adipocyte diameter.  Expression of HSL, ATGL and LPL. | | Increased body weight, TC, fat mass.  Improved adipocyte diameter.  Downregulated the expression of HSL and ATGL.  Upregulated the expression of LPL | | Xiang et al. (2018); Wei et al. (2019) |  |
|  |  | *In vitro* Human Hepatoma (HepG2) 37 cells. | | Cis-BF (10^-9^ to 10^-5^ M) for 2 h. | |  |  | Cell viability.  Intracellular triglyceride (TC) content.  PXR mRNA and protein expression levels of HepG2. | | cis-BF at 1×10^-5^ M reduced the viabilities to lower than 80%.  Increased PXR mRNA expression. | |  |  |
| LDL: Low density lipoproteins; LDL-R: low-density lipoprotein receptor; cAMP: cyclic adenosine monophosphate; P450scc: cytochrome P450 cholesterol side-chain cleavage enzyme; PEPCK: phosphoenol pyruvate carboxykinase; IC20: 20% inhibitory concentration; IC50: 50% inhibitory concentration; 3β-HSD: 3 beta-hydroxysteroid dehydrogenase; 17β-HSD: 17 betahydroxysteroid dehydrogenase; PR: Progesterone receptor; HLA-G: human leukocyte antigen G; CYPs: Cytochrome P450 enzymes; CYP17: cytochrome P450c17; CYP19: cytochrome P450c19; HSL: Hormone-sensitive lipase; TC: Total cholesterol; ATGL: Adipose triglyceride; LPL: Lipoprotein lipase; PXR: Pregnane X receptor; IP: Intraperitoneal; DEPs: Differentially expressed proteins; ALAT: alanine aminotransferase; ASAT: Aspartate aminotransferase; IL-1β: interleukin-1beta; TNF‑α: Tissue Necrotic Factor-α; IFN-γ: interferon-γ; MDA: Malondialdehyde; NO: Nitric oxide; GSH: reduced glutathione; GPx: glutathione peroxidase; SOD: Superoxide dismutase; CAT: Catalase; LDH: Lactate dehydrogenase; MFR: Mean firing rate; MBR: Mean burst rate; nAC: number of active channels; ROS: Reactive oxygen species; p.o: oral; i.v: intravenous; Nrf2: Nuclear factor erythroid-2; NF-kappaB: Nuclear factor43 kappaB pathway; PHA: Phytohemagglutinin; Bcl-2: B-cell lymphoma-2; JNK/MAPK: Jun N-terminal kinase/Mitogen-activated protein kinase; HSP: Heat shock protein; TFP: Trifluoropropenyl; NADPH: reduced Nicotinamide adenine dinucleotide phosphate; DA: Dopamine; DOPAC: 3,4-dihydroxyphenylacetic acid; HVA: Homovanillic acid; EPN: Epinephrine; NE: Norepinephrine; 5-HT: Serotonin; GR: Glucocorticoid receptor; ALP: Alanine phosphatase; TH: tyrosine hydroxylase | | | | | | | | | | | | | |

**Table S6.** Summary of human studies on bifenthrin exposure

| **Biological outcome** | **Study sample and location** | **Study design** | **Comparator** | **Main findings** | **References** |
| --- | --- | --- | --- | --- | --- |
| **BF disposition** | 53 Lactating mothers from Brazil, Columbia, and Spain. | Cross-sectional study. | Levels of BF (as well as other pyrethroids). | The average concentration of BF was detected in Brazil (Rondônia: 1.44 ng g−1 lw; São Paulo: 2.80 ng g−1 lw), Columbia (0.02 ng g−1 lw), and Spain (0.19). Differences between groups non-significant p>0.05). | Corcellas et al. (2012); Liang et al. (2022) |
|  | 132 Japanese children. |  | Urinary levels of BF metabolite. | Median daily intake =56 ng/kg b.w./d). Median urinary concentration of CTFA= 0.024 μg/g creatinine) with a detection frequency of 51%. |  |
| **Endocrine and metabolic effects** | Human studies: general Chinese population with a total sample size of 3822 participants. |  | Low/no levels of plasma BF. | Increase in FPG and a 5.19%, 10.49%, and 12.18% increase in FPI, HOMA-IR, and PCO levels. | Yoshida et al. (2021) |

**Table S7.** Risk of bias assessment for animal and in vitro studies on bifenthrin disposition and biological effects

|  | **Risk of bias assessment for animal and in vitro studies on BF disposition and biological effects** | | | | | | | | | |
| --- | --- | --- | --- | --- | --- | --- | --- | --- | --- | --- |
|  | **Selection bias** | | **Performance bias** | **Attrition/Exclusion analysis** | | **Detection bias** | | **Selective reporting bias** | **Other sources of bias** | |
| **Study** | **1. Was exposure level adequately randomised** | **2. Was allocation to the study groups adequately concealed** | **3. Were experimental conditions identical across study groups** | **4. We're research personnel blinded to the study groups during study** | **5. Were outcome data complete without attrition or exclusion from analysis** | **6. Can we be confident in the exposure characterization** | **7. Can we be confident in the outcome assessment** | **8. Were all measured outcomes reported** | **9. Were there any other potential threats to internal validity** | **Overall tier for RoB** |
| **Abdel-Wahhab et al., 2024a** | **+** | **+** | **+** | **+** | **-** | **++** | **++** | **++** | **--** | **1** |
| **Abdel-Wahhab et al., 2024b** | **+** | **+** | **++** | **--** | **-** | **-** | **+** | **++** | **--** | **2** |
| **Abdou et al., 2010** | **-** | **-** | **++** | **--** | **-** | **-** | **++** | **++** | **+** | **2** |
| **Bae et al., 2024** | **-** | **-** | **+** | **-** | **++** | **-** | **+** | **++** | **--** | **2** |
| **Bae and Kwon., 2021** | **-** | **-** | **+** | **-** | **++** | **-** | **+** | **++** | **--** | **2** |
| **Baska and Murthy, 2018** | **-** | **++** | **+** | **-** | **++** | **++** | **++** | **++** | **+** | **1** |
| **Beghoul et al., 2017** | **-** | **-** | **+** | **-** | **-** | **-** | **+** | **++** | **--** | **2** |
| **Bouaziz et al., 2020** | **-** | **-** | **+** | **-** | **-** | **-** | **+** | **++** | **-** | **2** |
| **Cao et al., 2014** | **-** | **-** | **++** | **-** | **-** | **++** | **+** | **++** | **+** | **1** |
| **Dar et al., 2013** | **++** | **+** | **++** | **-** | **+** | **+** | **++** | **++** | **+** | **1** |
| **Dar et al., 2019** | **++** | **+** | **++** | **-** | **+** | **+** | **++** | **++** | **+** | **1** |
| **Dar et al., 2015a** | **++** | **+** | **++** | **-** | **+** | **+** | **++** | **++** | **+** | **1** |
| **Dar et al., 2015b** | **++** | **+** | **++** | **-** | **-** | **+** | **++** | **++** | **-** | **1** |
| **Feriani et al., 2018** | **++** | **++** | **++** | **-** | **-** | **+** | **++** | **++** | **-** | **1** |
| **Gomaa et al., 2021** | **++** | **+** | **++** | **++** | **-** | **++** | **++** | **++** | **+** | **1** |
| **Gammon et al., 2015** | **++** | **++** | **++** | **+** | **++** | **++** | **++** | **++** | **+** | **1** |
| **Gammon et al., 2019** | **++** | **++** | **++** | **+** | **++** | **-** | **++** | **++** | **+** | **2** |
| **Gargouri et al., 2019** | **++** | **++** | **++** | **++** | **++** | **++** | **++** | **++** | **+** | **1** |
| **Gargouri et al., 2018a** | **++** | **++** | **++** | **-** | **++** | **++** | **++** | **++** | **+** | **1** |
| **Gargouri et al., 2018b** | **++** | **++** | **++** | **-** | **++** | **++** | **++** | **++** | **+** | **1** |
| **Gargouri et al., 2018c** | **+** | **++** | **++** | **-** | **++** | **++** | **++** | **++** | **-** | **1** |
| **Ham et al., 2020** | **-** | **++** | **++** | **-** | **++** | **++** | **++** | **++** | **-** | **1** |
| **Hoffman et al., 2006** | **+** | **+** | **+** | **-** | **-** | **-** | **-** | **++** | **--** | **2** |
| **Holton et al., 1997** | **-** | **-** | **+** | **-** | **-** | **-** | **+** | **++** | **--** | **2** |
| **Hughes and Edward, 2010** | - | **-** | **+** | **-** | **-** | **++** | **+** | **++** | **–** | **1** |
| **Hughes et al., 2016** | - | **-** | **+** | **-** | **-** | **++** | **+** | **++** | **–** | **2** |
| **Jin et al., 2012** | **++** | **-** | **++** | **-** | **+** | **++** | **+** | **++** | **--** | **1** |
| **Jin et al., 2014** | **+** | **+** | **+** | **-** | **+** | **-** | **+** | **++** | **--** | **2** |
| **Liu and Li, 2015** | **-** | **+** | **+** | **-** | **-** | **-** | **+** | **++** | **--** | **2** |
| **Liu et al., 2009** | **-** | **+** | **+** | **-** | **-** | **-** | **+** | **++** | **--** | **2** |
| **Liu et al., 2008** | **++** | **++** | **+** | **-** | **+** | **-** | **+** | **++** | **-** | **2** |
| **Liu et al., 2011** | **-** | **+** | **++** | **-** | **-** | **-** | **+** | **++** | **--** | **2** |
| **Lu, 2013** | **-** | **-** | **+** | **-** | **-** | **-** | **+** | **++** | **--** | **2** |
| **Lu et al., 2011** | **+** | **-** | **+** | **-** | **-** | **-** | **+** | **++** | **--** | **2** |
| **Mohana Krishnan and Prahkhya, 2016** | **-** | **-** | **+** | **-** | **++** | **++** | **+** | **++** | **-** | **2** |
| **Ortega et al., 2019** | **-** | **++** | **++** | **-** | **-** | **++** | **++** | **++** | **+** | **1** |
| **Mukhtar et al., 2022** | **-** | **+** | **++** | **-** | **-** | **-** | **+** | **++** | **-** | **2** |
| **Nallani et al., 2018** | **+** | **+** | **+** | **-** | **-** | **-** | **-** | **++** | **--** | **2** |
| **Pylak-Piwko and Nieradko-Iwanicka, 2021** | **++** | **+** | **++** | **-** | **+** | **-** | **-** | **++** | **--** | **2** |
| **Sadowska-Woda et al., 2010** | **-** | **++** | **++** | **-** | **++** | **+** | **+** | **++** | **--** | **1** |
| **Scollon et al., 2011** | **-** | **+** | **++** | **-** | **+** | **++** | **++** | **++** | **-** | **1** |
| **Shafer and Hughes, 2010** | **-** | **+** | **+** | **-** | **-** | **++** | **+** | **++** | **-** | **2** |
| **Syed et al., 2018** | **++** | **+** | **++** | **-** | **-** | **++** | **+** | **++** | **+** | **1** |
| **Syed et al., 2015b** | **++** | **+** | **++** | **-** | **-** | **++** | **+** | **++** | **+** | **1** |
| **Tatipamula and Kukavica, 2020** | **-** | **-** | **+** | **-** | **-** | **-** | **+** | **++** | **--** | **2** |
| **Tran et al., 2006** | **-** | **-** | **+** | **-** | **-** | **+** | **-** | **++** | **--** | **2** |
| **Wang et al., 2017** | **++** | **-** | **++** | **-** | **-** | **++** | **+** | **++** | **-** | **2** |
| **Wang et al., 2019** | **++** | **+** | **++** | **-** | **-** | **++** | **+** | **++** | **-** | **1** |
| **Wolansky et al., 2006** | **++** | **+** | **++** | **-** | **-** | **++** | **++** | **++** | **+** | **1** |
| **Wolansky et al., 2007** | **++** | **+** | **++** | **-** | **-** | **++** | **++** | **++** | **+** | **1** |
| **Xiang et al., 2018** | **-** | **++** | **+** | **-** | **-** | **-** | **+** | **++** | **-** | **2** |
| **Yang and Li, 2014** | **-** | **-** | **+** | **-** | **-** | **+** | **-** | **++** | **--** | **2** |
| **Yang et al., 2022** | **-** | **-** | **+** | **-** | **-** | **++** | **+** | **++** | **-** | **2** |
| **Zhang et al., 2018** | **-** | **-** | **+** | **-** | **-** | **-** | **+** | **++** | **-** | **2** |
| **Zhang et al., 2016** | **-** | **-** | **-** | **-** | **-** | **-** | **+** | **++** | **-** | **2** |
| **Zhang et al., 2015** | **++** | **-** | **-** | **-** | **-** | **++** | **+** | **++** | **-** | **2** |
| **Zhang and Zhang, 2024** | **++** | **-** | **++** | **-** | **-** | **++** | **+** | **++** | **-** | **1** |
| **Zhao et al., 2010** | **++** | **-** | **++** | **-** | **-** | **++** | **+** | **++** | **-** | **2** |
| **Zhao et al., 2014** | **-** | **-** | **-** | **-** | **-** | **++** | **+** | **++** | **-** | **2** |

| **Risk of bias rating levels** | |
| --- | --- |
| **Definitely low risk** | **++** |
| **Probably low risk** | **+** |
| **Probably high risk** | **-** |
| **Definitely high risk** | **- -** |

**Table S8.** Risk of bias assessment for human studies on BF disposition and biological effects

|  | **Risk of bias assessment for human studies on BF disposition and biological effects** | | | | | | | |
| --- | --- | --- | --- | --- | --- | --- | --- | --- |
|  | **Selection bias** | **Confounding variables** | **Attrition/ Exclusion** | **Detection bias** | | **Selective reporting bias** | **Other sources of bias** | |
| **Study** | **1. Did selection of study participants result in appropriate comparison groups** | **2. Did the study design or analysis account for any confounding and modifyng variables** | **3. Were outcome data complete without attrition or exclusion from analysis** | **4. Was BF and/or its metabolites measured in an human sample** | **5. Can we be confident in the outcome assessment** | **6. Were all measured outcomes reported** | **7. Were there any other potential threats to internal validity** | **Overall risk tier** |
| **Corcellas et al., 2012** | **++** | **-** | **-** | **++** | **+** | **++** | **-** | **2** |
| **Liang et al., 2022** | **++** | **++** | **-** | **++** | **++** | **++** | **+** | **1** |
| **Yoshida et al., 2021** | **+** | **-** | **--** | **++** | **+** | **++** | **-** | **2** |

| **Risk of bias rating levels** | |
| --- | --- |
| **Definitely low risk** | **++** |
| **Probably low risk** | **+** |
| **Probably high risk** | **-** |

**Table S9:** Summary of oral, dermal, inhalation, and eye toxicity studies

| **Study** | **Result Summary** | **Reference** |
| --- | --- | --- |
| **Metabolism and toxicokinetic** | | |
| Rat | Most (>70%) of the administered radioactivity was found in faces and about 20% in urine. It has a slow GI absorption and elimination rate, low bioaccumulation except in fat stores, and a metabolic route mainly through the hydrolysis of the ester linkage. It is mainly excreted in the feces. | USEPA, 2020, FAO, 2009 |
| **Acute toxicity** | | |
| Acute oral (rodent) | LD_50_ = 43.0 - 58.4 mg/kg  LD_50_ = 168 - 211 mg/kg  Toxicity category I | USEPA, 2020; ECHA, 2009 |
| Acute dermal (rat) | LD_50_ ≥ 2000 mg/kg (males and females).  Toxicity category III | USEPA, 2020; ECHA, 2009 |
| Acute inhalation (rat) | LC_50_ = 0.8 - 1.10 mg/L  Toxicity category III | USEPA, 2020; ECHA, 2009 |
| Eye and Skin irritation | Conjunctivitis in 3/3 eyes resolving on day 4  Moderate/no irritant  Dermal sensitizer  Toxicity category III (eye) and IV (skin). | USEPA, 2020; ECHA, 2009 |
| Acute oral toxicity in Long Evans rats | BMDL_1SD_ = 3.1 mg/kg;  BMD_1SD_ = 4.1 mg/kg based on decreased motor activity. | Special study (Wolansky et al., 2006) |
| Acute oral toxicity in rats | BMDL_1SD_ = 0.4 mg/kg; BMD_1SD_ = 14.3 mg/kg based on multiple functional observatory battery changes. | Special study (Weiner et al., 2009) |
| **Subchronic and chronic** | | |
| 28-day oral toxicity in rat | NOAEL = 11 mg/kg bw/day; LOAEL = 22 mg/kg bw/day | ECHA, 2009 |
| 90-day oral toxicity | **Rat**  NOAEL = 3 - 5 mg/kg/day; LOAEL = 7 - 9 mg/kg/day  **Dog**  NOAEL = 2.0 - 2.5 mg/kg/day;  LOAEL = 4 - 5 mg/kg/day | USEPA, 2020, ECHA, 2009 |
| Chronic toxicity (1 year) in dog | NOAEL= 1.3 – 1.5 mg/kg/day;  LOAEL= 2.7 – 3.0 mg/kg/day based on increased incidence of tremors. | USEPA, 2020, ECHA, 2009 |
| Combined chronic toxicity/carcinogenicity (1-2 year) in dog | NOAEL= 2 -3 mg/kg/day;  LOAEL= 4.5 – 6.5 mg/kg/day based on increased incidence of tremors.  No conclusive evidence of carcinogenic potential. | USEPA, 2020, ECHA, 2009 |
| Carcinogenicity (1-2 years) in mice | NOAEL= 6.5 – 9.0 mg/kg/day;  LOAEL= 25.5 – 33.0 mg/kg/day based on increased incidence of tremors. | USEPA, 2020; ECHA, 2009, FAO, 2009 |
| Inhalation toxicity in rat | NOAEL = 0.0059 mg/L/day  LOAEL= 0.0196 mg/kg/day; based on increased tremors and increased respiration rate. | USEPA, 2020 |
| Dermal toxicity | **Rabbit**  NOAEL= 88 mg ai/kg/day; LOAEL= 442 mg ai/kg/day based on loss of muscle coordination and increased incidence of tremors.  **Rat**  NOAEL= 47 mg ai/kg/day; LOAEL=93 mg ai/kg/day based on staggered gait (males) and exaggerated hind limb flexion. | USEPA, 2020 |

**Table S10:** Mutagenicity in vitro tests

| **Species** | **Test/conditions** | **Result** | **Reference** |
| --- | --- | --- | --- |
| *Salmonella typhimurium* | Ames Assay | Not mutagenic | USEPA, 2020, FAO, 2009, ECHA, 2009 |
| Mouse Lymphoma Cells | Mouse Lymphoma Mutagenesis (with and without metabolic actication). | Weak positive results with and without metabolic activation. | USEPA, 2020, FAO, 2009, ECHA, 2009 |
|  | HGPRT Gene Mutation | Not mutagenic |  |
| Rat (M) | *In vivo* Cytogenetics | Negative | FAO, 2009, ECHA, 2009 |
| Chinese Hamster Ovary  (CHO) (F) | *In vitro* Chromosome Aberration with and without activation | Negative | USEPA, 2020, FAO, 2009, ECHA, 2009 |
|  | HGPRT Assay (with and without S9) | Inconclusive w/metabolic activation |  |
|  | *In vitro* Gene Mutation  *In vitro* Sister Chromatid Exchange | Not mutagenic |  |
| Rat hepatocytes | Unscheduled DNA Synthesis | Marginally positive at one highly toxic dose. Two repeat assays yielded negative responses. | USEPA, 2020, FAO, 2009, ECHA, 2009 |
| Mouse embryo cells (BALB/3T3) | Cell Transformation | Negative | FAO, 2009, ECHA, 2009 |
| Drosophila | Sex Linked Recessive Lethal – Genotox | Negative | FAO, 2009, ECHA, 2009 |

**Table S11**: Neurotoxicity studies

| **Study** | **Result Summary** | **Reference** |
| --- | --- | --- |
| **Acute toxicity** | | |
| Acute neurotoxicity in rats | NOAEL= 35 mg/kg/day;  LOAEL= 75 mg/kg/day based on mortality (females only), clinical and FOB findings, and motor activity. | USEPA, 2020 |
| Subchronic neurotoxicity in rats | NOAEL= 2.9-4.0 mg/kg/day  LOAEL= 6.0 – 7.5 mg/kg/day based on neuromuscular findings such as grip strength, tremors, and landing foot splay). | USEPA, 2020, ECHA, 2009 |
| Developmental neurotoxicity in rats | **Maternal**  NOAEL= 3.5 – 8.5 mg/kg/day (during lactation and gestation)  LOAEL= 7.0 – 16.5 mg/kg/day based on clinical signs of neurotoxicity (during lactation and gestation).  **Developmental**  NOAEL= 3.0 -7.5 mg/kg/day (lactation and gestation)  LOAEL= 7.0 – 16.5 mg/kg/day. | USEPA, 2020, ECHA, 2009, FAO, 2009 |

**Table S12:** Endocrine studies

| **Hormone** | **Lines of evidence** | **Results summary** | **Reference** |
| --- | --- | --- | --- |
| Estrogen pathway | ER binding, ER activation/gene expression, steroidogenesis, sex steroid hormone, uterine weight, ovarian weight, ovarian/gonadal staging, pituitary weight, Estrous cyclicity, Age/weight at VO, 2° sex characteristics, fertility, vitellogenin, systemic toxicity observed, overt toxicity observed. | Negative for most EDSP tier 1 assays.  ER binding was positive for cell proliferation and gene expression in non-guideline studies considered for analysis. | USEPA, 2015 |
| Androgen pathway | AR binding, steroidogenesis, sex steroid hormone, testes weight, epididymis weight, epididymis histopathology, pituitary weight, 2° sex characteristics, Age and weight at PPS, vitellogenin, systemic toxicity observed, overt toxicity observed. | Negative for most EDSP tier 1 assays, while a few were not examined or reported.  Some signs of overt toxicity were observed. |  |
| Thyroid hormone pathway | Thyroid weight, gross histopathology, Serum T_4_ levels, TSH levels, pituitary weight, developmental stage, growth, systemic and overt toxicity. | Negative for most EDSP tier 1 assays.  Some signs of systemic and overt toxicity were observed at high treatment. |  |

EDSP Tier 1 assays: ER Binding Assay (Rat uterine cytosol), ERα Transcriptional Activation Assay (Human cell line HeLa 9903), AR Binding Assay (Rat prostate cytosol), Steroidogenesis Assay (Human cell line H295R), Aromatase Assay (human recombinant microsomes), Uterotrophic Assay (Rat), Hershberger Assay (Rat), Pubertal Female Assay (Rat), Pubertal male Assay (Rat), Fish Short-term Reproduction Assay, Amphibian Metamorphosis Assay (Frog).

**Table S13:** Reproductive studies

| **Test** | **Result Summary** | **Study identifier** |
| --- | --- | --- |
| Developmental toxicity (gavage) (rat)  Range-finding study | **Maternal**  NOAEL= 0.88 mg/kg/day  LOAEL= 1.77 mg/kg/day based on tremors during gestation  **Developmental**  NOAEL= NOAEL not determined (fetuses not examined)  LOAEL= LOAEL not determined (fetuses not examined)  Acceptable-guideline | USEPA, 2020 |
| Developmental toxicity (dietary) (rat). | **Maternal**  NOAEL= 7.1 mg/kg/day  LOAEL= 15.5 mg/kg/day based on clinical signs, decreased food consumption and body weight gains adjusted for gravid uterine weight.  **Developmental**  NOAEL= 15.5 mg/kg/day  LOAEL= LOAEL (not observed)  Acceptable-guideline | USEPA, 2020 |
| Developmental toxicity in rabbit. | **Maternal**  NOAEL= 2.36 mg/kg/day  LOAEL= 3.5 mg/kg/day based on treatment-related head and forelimb twitch.  **Developmental**  NOAEL= greater than 7 mg/kg/day  LOAEL= LOAEL (not observed)  Acceptable-guideline | USEPA, 2020 |
| Multigeneration reproductive toxicity in rats | **Parental/systemic toxicity**  NOAEL= 5.0 mg/kg/day (males) and 3.0 mg/kg/day (female)  LOAEL= 5.0 mg/kg/day (females) based on tremors and decreased body weight; not observed for males.  **Reproductive/offspring toxicity**  NOAEL= 5.0 mg/kg/day  LOAEL= not observed | USEPA, 2020 |
| Rat study | **Maternal**  NOAEL = 1 mg/kg  **Teratogenicity and embryogenicity**  NOAEL = >2 mg/kg | ECHA, 2009 |

**Table S14**: Non-cancer exposure and risk assessment for registered uses of bifenthrin in the US

| **Exposure type** | **Exposure scenario** | **Risk estimates** | **Evaluation of risk concern** | **Reference** |
| --- | --- | --- | --- | --- |
| Dietary | Food and drinking water | < 100% aPAD for all populations | No risk estimates of concern.  Short-term exposure concern was identified for strawberries in Europe.  Acute dietary exposure to BF residues from the consumption of strawberries may present a public health concern. | USEPA, 2020; EFSA, 2023 |
|  |  | **Acute exposure**  Max 125% ARfD for strawberries based (the EU ARfD scenario)  Max 376% ARfD (the JMPR scenario)  Short-term exposure:  Highest result for children:  380% of ARfD  **Chronic exposure**  Overall was 43% ADI with the strawberry contribution accounting for up to 1.5% of the ADI (the EU ADI scenario)  Overall was 64% ADI (the JMPR ADI scenario)  Long-term  exposure: Max 10–40% of  the JMPR ADI. |  |  |
| Residential | Handling and post-application: Lawns, indoor environments, gardens, trees, and pets. | ARIs (dermal + inhalation) > LOC of 1 | No risk estimates of concern for (1)episodic granular ingestion following granular application to lawns/turf (MOE = 85, LOC = 100) assuming the maximum % ai in registered granular formulations of BF (0.2%) a maximum application rate of 200 lb.  Product/A (0.50 lb ai/A), and (2) ingestion rates adjusted for BF-specific application rates. This scenario is not of concern (MOE=100) when assuming a maximum application rate of 170 lbs product/A (0.34 lb ai/A). |  |
| Occupational handler | Majorly crop application | Dermal MOEs ≥ 100, inhalation MOEs ≥ 30, and ARI ≥ 1. | No risk estimate of concern except Mixing/Loading liquids for aerial ultra-low volume (ULV) application to cotton (Dermal MOE = 79, Inhalation MOE = 510, ARI = 0.75). |  |
| Non-Occupational Spray Drift Exposure | Spray drift | MOEs > LOC of 100 | No risk estimate of concern |  |

aPAD: acute population adjusted dose; MOE: Margin of exposure; LOC: Level of concern; ARI=Aggregate risk index; ARfD: Acute reference dose; EU: European Union; JMPR: FAO/WHO Joint Meeting on Pesticide Residues; ADI: Acute dietary intake

**NB:** Details on regulatory guideline toxicity and risk assessment data can be retrieved from

USEPA: https://downloads.regulations.govEPA-HQ-OPP-2016-0352-0008/content.pdf

ECHA: https://echa.europa.eu/documents/10162/81b13962-33ca-4a28-81fa-69c3c0e11a0f

FAO/WHO: https://extranet.who.int/pqweb/sites/default/files/vcp-documents/WHOVC-SP_BF_2022.pdf

**References**

[ECHA] European Chemical Agency. *Bifenthrin: CLH report*. <https://echa.europa.eu/documents/10162/81b13962-33ca-4a28-81fa-69c3c0e11a0f>

[EU] European Commission. (2012a). Commission Implementing Regulation (EU) No 582/2012 of 2 July 2012 approving the active substance bifenthrin in accordance with Regulation (EC) No 1107/2009 of the European Parliament and of the Council concerning the placing of plant protection products on the market, and amending the Annex to Commission Implementing Regulation (EU) No 540/2011. *Official Journal of the European Union, L 173*, 3–7.

[EU] European Commission. (2012b). Commission Regulation (EU) No 441/2012 of 24 May 2012 amending Annexes II and III to Regulation (EC) No 396/2005 of the European Parliament and of the Council as regards maximum residue levels for bifenazate, bifenthrin, boscalid, cadusafos, chlorantraniliprole, chlorothalonil, clothianidin, cyproconazole, deltamethrin, dicamba, difenoconazole, dinocap, etoxazole, fenpyroximate, flubendiamide, fludioxonil, glyphosate, metalaxyl-M, meptyldinocap, novaluron, thiamethoxam, and triazophos in or on certain products. *Official Journal of the European Union, L 135*, 4–56.

[EU] European Commission. (2017). Commission Regulation (EU) 2017/170 of 30 January 2017 amending Annexes II, III and V to Regulation (EC) No 396/2005 of the European Parliament and of the Council as regards maximum residue levels for bifenthrin, carbetamide, cinidon-ethyl, fenpropimorph and triflulsulfuron in or on certain products. *Official Journal of the European Union, L 30*, 1–44.

[EU] European Commission. (2018). Commission Regulation (EU) 2018/687 of 4 May 2018 amending Annexes II and III to Regulation (EC) No 396/2005 of the European Parliament and of the Council as regards maximum residue levels for acibenzolar-S-methyl, benzovindiflupyr, bifenthrin, bixafen, chlorantraniliprole, deltamethrin, flonicamid, fluazifop-P, isofetamid, metrafenone, pendimethalin and teflubenzuron in or on certain products. *Official Journal of the European Union, L 121*, 63–104.

[EU] European Commission. (2008). *Regulation (EC) No 1272/2008 of the European Parliament and of the Council of 16 December 2008 on classification, labelling, and packaging of substances and mixtures, amending and repealing Directives 67/548/EEC and 1999/45/EC, and amending Regulation (EC) No 1907/2006*.

[EU] European Commission. (2018). *Commission Regulation (EU) 2018/605 of 19 April 2018 amending Annex II to Regulation (EC) No 1107/2009 by setting out scientific criteria for the determination of endocrine disrupting properties*.

[FAO] Food and Agriculture Organization of the United Nations. (2022). *Bifenthrin: Specifications and evaluations for public health pesticides*. <https://extranet.who.int/pqweb/sites/default/files/vcp-documents/WHOVC-SP_BF_2022.pdf>

Baskar, M. K., & Murthy, P. B. (2018). Acute in vitro neurotoxicity of some pyrethroids using microelectrode arrays. *Toxicology in Vitro, 47*, 165–177. <https://doi.org/10.1016/j.tiv.2017.11.010>

Dar, M. A., Khan, A. M., Raina, R., Beigh, S. A., & Sultana, M. (2015b). Effect of repeated oral administration of bifenthrin antioxidant status and acetylcholinesterase activity in brain of rats. *Toxicological and Environmental Chemistry, 97*(7), 961–967. <https://doi.org/10.1080/02772248.2015.1070160>

Dar, M. A., Raina, R., Mir, A. H., Sultana, M., & Pankaj, N. K. (2015a). Effect of dermal application of bifenthrin on acetylcholinesterase and oxidative stress indices in rat blood, lung and kidney. *Proceedings of the National Academy of Sciences, India, Section B: Biological Sciences, 85*, 431–435. <https://doi.org/10.1007/s40011-014-0390-z>

Dar, M. A., Khan, A. M., Raina, R., Verma, P. K., & Sultana, M. (2013). Effect of repeated oral administration of bifenthrin on lipid peroxidation and antioxidant parameters in Wistar rats. *Bulletin of Environmental Contamination and Toxicology, 91*(1), 125–128. <https://doi.org/10.1007/s00128-013-1022-7>

Liu, H., Xu, L., Zhao, M., Liu, W., Zhang, C., & Zhou, S. (2009). Enantiomer-specific, bifenthrin-induced apoptosis mediated by MAPK signaling pathway in Hep G2 cells. *Toxicology, 261*(3), 119–125. <https://doi.org/10.1016/j.tox.2009.05.002>

Liu, J., Yang, Y., Zhuang, S., Yang, Y., Li, F., & Liu, W. (2011b). Enantioselective endocrine-disrupting effects of bifenthrin on hormone synthesis in rat ovarian cells. *Toxicology, 290*(1), 42–49. <https://doi.org/10.1016/j.tox.2011.08.016>

Lu, X. (2013). Enantioselective effect of bifenthrin on antioxidant enzyme gene expression and stress protein response in PC12 cells. *Journal of Applied Toxicology, 33*(7), 586–592. <https://doi.org/10.1002/jat.1774>

Mukhtar, F., Jilani, K., Bibi, I., Mushataq, Z., Bari Khan, M. A., & Fatima, M. (2022). Stimulation of erythrocyte membrane blebbing by bifenthrin induced oxidative stress. *Dose-Response, 20*(1), 15593258221076710. <https://doi.org/10.1177/15593258221076710>

Ortega, M. E. M., Pato, A. M., Romero, D. M., Holt, C. S. S., Alvarez, G., Ridolfi, A., ... Wolansky, M. J. (2019). Relationship between the dose administered, target tissue dose, and toxicity level after acute oral exposure to bifenthrin and tefluthrin in young adult rats. *Toxicological Sciences, 172*, 225–234. <https://doi.org/10.1093/toxsci/kfz204>

Patty, F. A. (2001). *Industrial hygiene and toxicology* (5th ed., G. D. Clayton & F. E. Clayton, Eds.). Wiley.

Sadowska-Woda, I., Popowicz, D., & Karowicz-Bilińska, A. (2010). Bifenthrin-induced oxidative stress in human erythrocytes in vitro and protective effect of selected flavonols. *Toxicology in Vitro, 24*(2), 460–464. <https://doi.org/10.1016/j.tiv.2009.09.024>

Scollon, E. J., Starr, J. M., Crofton, K. M., Wolansky, M. J., DeVito, M. J., & Hughes, M. F. (2011). Correlation of tissue concentrations of the pyrethroid bifenthrin with neurotoxicity in the rat. *Toxicology, 290*, 1–6.

Shafer, T. J., & Hughes, M. F. (2010). Accumulation of pyrethroid compounds in primary cultures from rat cortex. *Toxicology in Vitro, 24*(7), 2053–2057. <https://doi.org/10.1016/j.tiv.2010.08.016>

Zhang, L., Zuo, Y., Liu, H., & Wang, X. (2020). Study on the solid–liquid equilibrium of bifenthrin in solvents. *Journal of Molecular Liquids, 308*, 113033. <https://doi.org/10.1016/j.molliq.2020.113033>

**Figure (attached as a separate file)**

**Figure S1.** The main target of BF's toxic action is the cell membrane. There is a change in the transmembrane flux of Na^+^, Ca^2+^, Cl^-^, and K^+^, which may be related to symptoms of depression. Exposure to BF leads to oxidative stress.
